# Commensal microbiota induces colonic barrier structure and functions that contribute to homeostasis

**DOI:** 10.1038/s41598-018-32366-6

**Published:** 2018-09-21

**Authors:** Christina L. Hayes, Jasmine Dong, Heather J. Galipeau, Jennifer Jury, Justin McCarville, Xianxi Huang, Xuan-Yu Wang, Avee Naidoo, Arivarasu N. Anbazhagan, Josie Libertucci, Conor Sheridan, Pradeep K. Dudeja, Dawn M. E. Bowdish, Michael G. Surette, Elena F. Verdu

**Affiliations:** 1 Farncombe Family Digestive Health Research Institute, McMaster University, Hamilton, Ontario Canada; 2 Department of Pathology and Molecular Medicine, McMaster University, Hamilton, Ontario Canada; 3 Institute for Infectious Diseases Research, McMaster University, Hamilton, Ontario Canada; 4 Division of Gastroenterology and Hepatology, Department of Medicine, University of Illinois at Chicago, Chicago, Illinois USA; 5 Jesse Brown VA Medical Centre, University of Illinois at Chicago, Chicago, Illinois USA; 6 Department of Biochemistry and Biomedical Sciences, McMaster University, Hamilton, Ontario Canada

## Abstract

The intestinal barrier encompasses structural, permeability and immune aspects of the gut mucosa that, when disrupted, may contribute to chronic inflammation. Although gnotobiotic studies have demonstrated the effects of microbiota on mucosal and systemic immunity, as well as intestinal barrier architecture and innate immune characteristics, its impact on barrier function remains unclear. We compared germ-free and conventional mice, as well as mice colonized with human fecal microbiota that were followed for 21 days post-colonization. Colonic barrier structure was investigated by immunohistochemistry, molecular and electron microscopy techniques. Permeability was assessed in colon tissue by Ussing chambers, and by serum LPS and MDP detection using TLR4- and NOD2-NFκB reporter assays. Microbiota profile was determined by Illumina 16S rRNA gene sequencing. Low dose dextran sodium sulfate was administered to assess microbiota-induced barrier changes on resistance to colonic injury. Permeability to paracellular probes and mucus layer structure resembled that of conventional mice by day 7 post-colonization, coinciding with reduced claudin-1 expression and transient IL-18 production by intestinal epithelial cells. These post-colonization adaptations were associated with decreased systemic bacterial antigen exposure and reduced susceptibility to intestinal injury. In conclusion, commensal colonization promotes physiological barrier structural and functional adaptations that contribute to intestinal homeostasis.

## Introduction

Host-microbe interactions are key determinants of health and disease through their influence on host physiology. Gnotobiotic studies have demonstrated that the microbiota impacts immune development and function, metabolism, intestinal morphology and motility, as well as the gut-brain axis^1–8^. One important functional parameter that has been implicated in homeostasis as well as disease induction is the intestinal barrier. This is an all-encompassing term given to the physical and chemical barrier that makes up the first line of defense against luminal antigens and pathogens. Influenced by interactions with mucosal immune cells and the intestinal microbiota, this contiguous layer of epithelial cells (ECs) determines intestinal permeability, mucus production, and secretion of immune factors, such as antimicrobial peptides. Previous studies have shown that the microbiota influences intestinal barrier architecture, mucus layer, as well as innate immune factors, such as pattern recognition receptor expression and antimicrobial peptide production, although the effects on barrier permeability remain poorly characterized^3,9,10^. Intestinal barrier dysfunction and altered microbiota composition have been associated with chronic inflammatory gastrointestinal conditions, such as inflammatory bowel disease^11–13^. However, deciphering the role of dysfunctional microbiota-barrier interactions during the course of disease has proven difficult, particularly since there is little understanding of how the microbiota impacts the intestinal barrier in the non-inflamed, physiological state.

We therefore studied intestinal structure and permeability in germ-free (GF) and conventionally raised, specific pathogen-free (SPF) mice. The dynamics of structural and permeability adaptations induced were assessed over a 21-day period following *de novo* colonization of GF mice with human fecal microbiota from a healthy donor previously used in a successful fecal microbiota transfer clinical trial^14^. In order to evaluate the pathophysiological consequences of colonization-induced barrier changes, resistance towards dextran sulfate sodium (DSS)-induced injury was assessed. We show that microbial colonization with commensal microbiota induced rapid colonic barrier structural and permeability functions important for maintenance of homeostasis.

## Results

### The microbiota is a key determinant of a physiological colonic barrier

**Figure 1:**
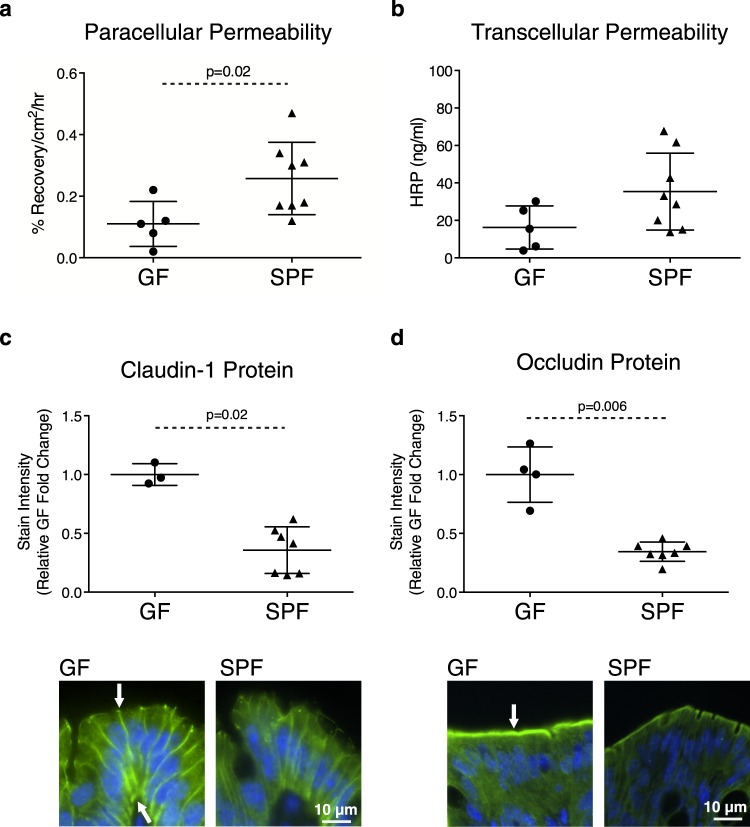
Microbiota is required for establishment of physiological colonic barrier permeability. (**a**) Paracellular permeability to ^51^Cr-EDTA and (**b**) transcellular permeability to HRP in the proximal colon. Evaluation of (**c**) claudin-1 and (**d**) occludin protein expression by IF and (**e**) representative images. Target proteins are shown in green, nuclei were stained with DAPI (blue). Arrows indicate areas of high expression. Each data point represents one mouse; the horizontal line and whiskers depict the mean ± standard deviation (SD). Data was collected over 2–5 independently executed experiments. Significance was determined using an unpaired, two-tailed Mann-Whitney test.

**Figure 2:**
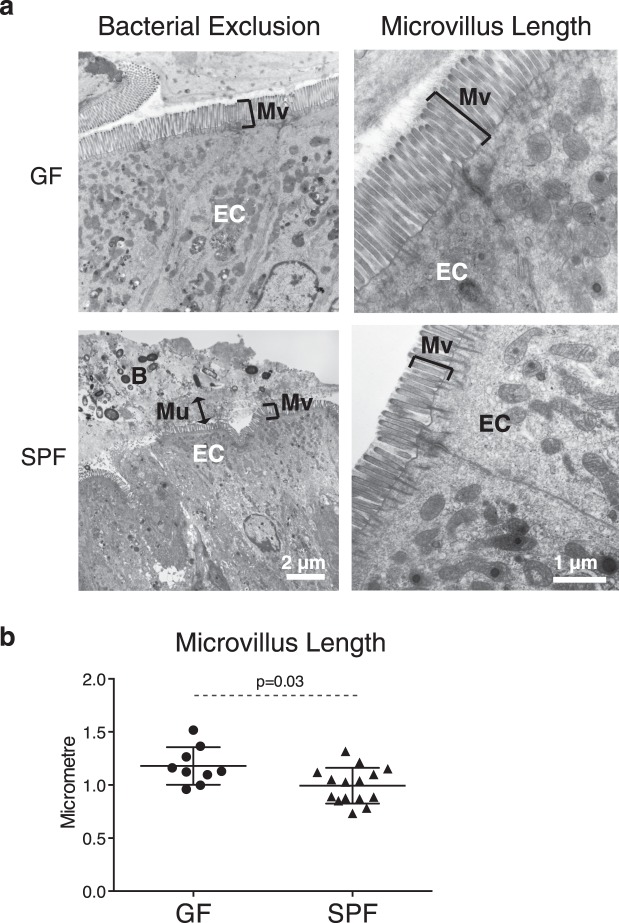
Microbiota is required for establishment of physiological colonic barrier mucus and microvillus structure. Mucus structure and microvillus length were evaluated in GF and SPF mice. (**a**) Representative EM pictures demonstrating bacterial exclusion and microvillus length. Markings indicate microvilli (Mv), epithelial cells (EC), and the black arrow indicates separation of bacteria (B) from the epithelium by the mucus (Mu) layer. (**b**) Microvillus length. Each data point represents analysis of one image; the horizontal line and whiskers depict the mean ± SD. Data was collected over 2–5 independently executed experiments. Significance was determined using an unpaired, two-tailed Mann-Whitney test.

We first compared colonic barrier permeability and structure between GF and conventional SPF mice, whereby characteristics observed in SPF mice were considered the standard, physiological state. Paracellular permeability to the probe ^51^Cr-EDTA was lower in the proximal colon of GF mice compared with SPF mice (Fig. 1a). No statistically significant changes in transcellular permeability to horseradish peroxidase (HRP) was observed (Fig. 1b). Semi-quantification of TJ protein expression by immunofluorescence (IF) revealed higher expression of claudin-1 and occludin in GF mice compared to SPF mice (Fig. 1c,d). No significant change in ZO-1 protein was observed (Supplementary Fig. S1). Tight junction (TJ) mRNA expression by real-time (RT)-qPCR showed GF mice had lower claudin-1 and higher occludin mRNA expression compared with SPF mice (Supplementary Fig. S1). A non-statistically significant trend for higher ZO-1 mRNA expression was also found in GF mice. To further assess the impact of microbiota on barrier function, expression of apical membrane transporters was evaluated. No significant differences in serotonin transporter (SERT), apical sodium bile acid transporter (ASBT), cystic fibrosis transmembrane conductance regulator (CFTR), downregulated-in-adenoma (DRA), monocarboxylate transporter 1 (MCT-1), Niemann-Pick C1-like protein 1 (NPC1L1), sodium-hydrogen exchanger 3 (NHE3), or sodium-coupled monocarboxylate transporter (SMCT-1) expression were observed between GF and SPF mice (Supplementary Fig. S2). Electron microscopy (EM) assessment demonstrated the glycocalyx of the mucus layer in SPF mice excluded bacteria from the epithelial surface, while no bacteria were observed in EM of GF mice (Fig. 2a). GF mice also had higher microvilli length compared with SPF mice (Fig. 2b). These findings indicate that the presence of commensal microbiota impacts colonic ultrastructure, TJ proteins and paracellular permeability.

### Colonic microbiota taxonomic structure changes over the first week of colonization

**Figure 3:**
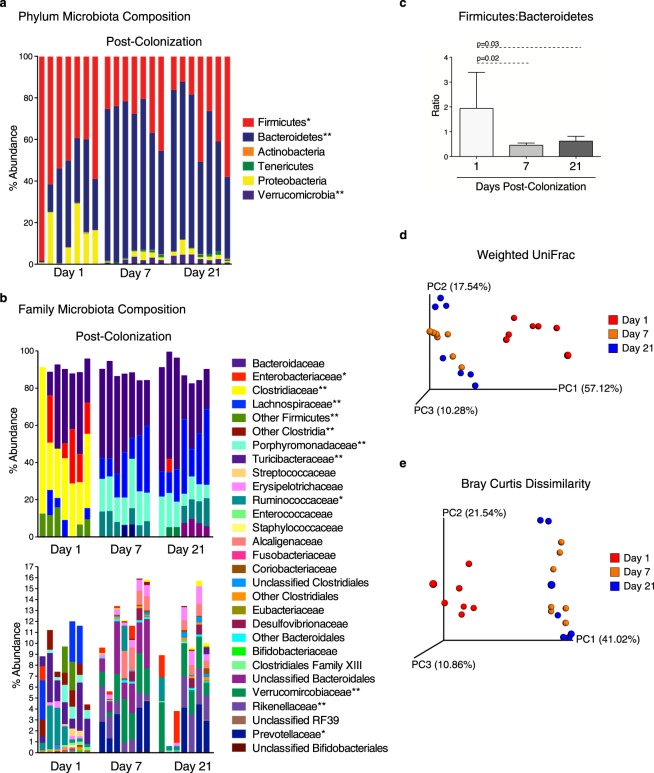
Microbiota taxonomic composition shifts between days 1 and 7 post-colonization. Fecal microbiota was assessed by 16S rRNA gene sequencing of the same 7 mice at days 1, 7 and 21 post-colonization. Fecal microbiota taxonomic structure at the (**a**) phylum and (**b**) family levels. *p < 0.05; **p < 0.01 as determined by Kruskal-Wallis test with Bonferroni correction. (**c**) Firmicutes:Bacteroidetes ratio (n = 7 per group). Error bars depict the SD. Significance was determined by Kruskal-Wallis test with Dunn’s post-hoc test. (**d**) Principle component (PC) analysis using weighted UniFrac and (**e**) Bray Curtis dissimilarity ordination. Each data point represents one mouse. Data was collected in a single experiment.

In order to evaluate the dynamics of intestinal barrier changes after colonization, GF mice were colonized with human microbiota from one healthy donor previously shown to be free of common pathogens (Supplementary Fig. S3)^14^. The fecal bacterial profile of mice colonized with this donor was monitored over the first 21 days of colonization by 16S rRNA gene sequencing. Fecal bacterial composition shifted over the first week post-colonization (Fig. 3a,b); compared to days 7 and 21 post-colonization, a high abundance of Firmicutes with significantly more Turicibacteraceae, Clostridiaceae, other Clostridia, and other Firmicutes was observed at day 1 post-colonization (p < 0.05 days 7 and 21 post-colonization vs day 1). By day 7 post-colonization, expansion of Verrucomicrobia and Bacteroidetes was observed, with other Bacteroidales, Porphyromonadaceae, Rikenellaceae, and Verrucomicrobiaceae significantly increased compared to day 1 (p < 0.05 days 7 and 21 post-colonization vs day 1). Overall, the Firmicutes:Bacteroidetes ratio was significantly higher at day 1 post-colonization compared to days 7 and 21 post-colonization (Fig. 3c). Principal component analysis with weighted UniFrac and Bray Curtis dissimilarity ordinations showed the microbiota at day 1 post-colonization clustered separately from days 7 and 21 (Fig. 3d,e). Although no statistically significant changes in taxonomic composition were observed between days 7 and 21 post-colonization, Bray Curtis dissimilarity values between mice at day 21 post-colonization were higher than at days 1 and 7 post-colonization, indicating higher interindividual variation (Supplementary Fig. S4). When comparing Bray Curtis dissimilarity values of each individual mouse post-colonization, the values were lower between days 7 and 21 post-colonization compared to day 1 vs 7 and day 1 vs 21. No significant changes in Shannon diversity, PD whole tree, Chao1 or observed species were found (Supplementary Fig. S5). Together, these findings indicate changes in the taxonomic composition of the fecal microbiota occur over the first 21 days post-colonization.

### Colonization induces physiological levels of colonic paracellular permeability

**Figure 4:**
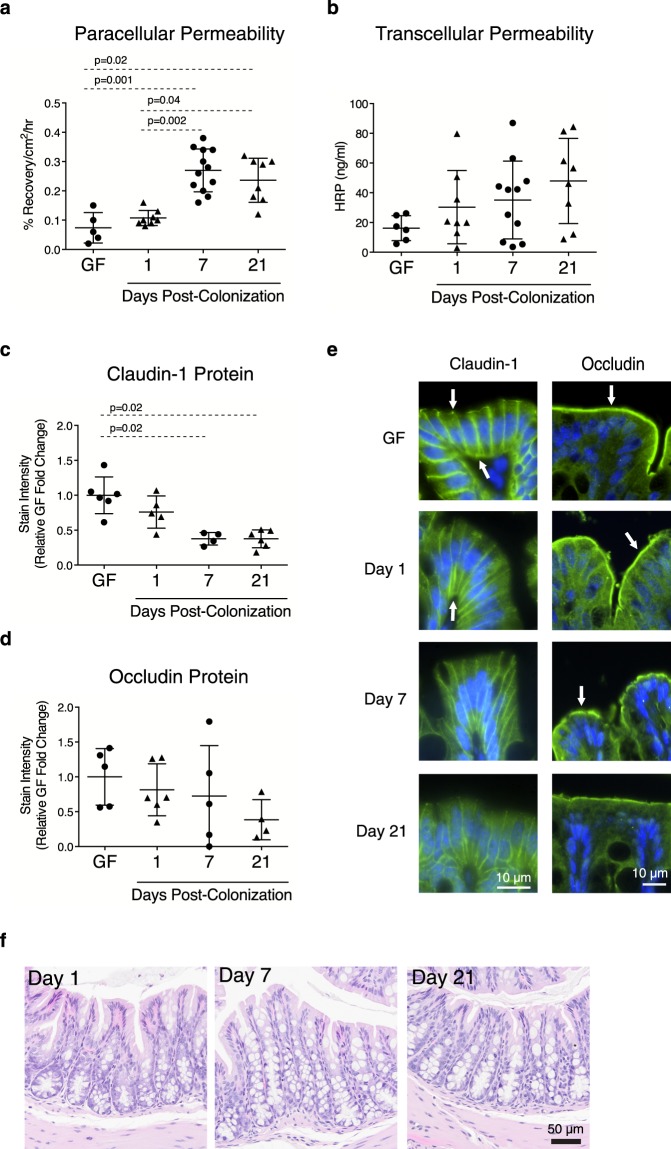
Microbiota induces maturation of colonic permeability. Colonic permeability as well as TJ mRNA and protein expression were assessed at days 1, 7 and 21 post-colonization. (**a**) Paracellular permeability to ^51^Cr-EDTA and (**b**) transcellular permeability to HRP. (**c**) Evaluation of claudin-1 and (**d**) occludin protein expression by IF. (**e**) Representative IF images of TJ proteins. Target proteins are shown in green, nuclei were stained with DAPI (blue). Arrows indicate areas of high expression. (**f**) Representative images of colon mucosal structure. Each data point represents one mouse; the horizontal line and whiskers depict the mean ± SD. Data was collected over 2–5 independently executed experiments. Significance was determined by Kruskal-Wallis test with Dunn’s post-hoc test.

By day 7 post-colonization, uptake of the paracellular probe ^51^Cr-EDTA increased to a level comparable to that observed in SPF mice, and this was maintained up to day 21 post-colonization (Fig. 4a). As with SPF mice, no changes in transcellular permeability were observed post-colonization (Fig. 4b). The increase in paracellular probe uptake coincided with lower expression of claudin-1 protein at days 7 and 21 post-colonization (Fig. 4c,d). Occludin protein expression tended to be lower by day 21 post-colonization, but did not reach statistically significance (Fig. 4e,d). Only ZO-1 mRNA expression was diminished at day 1 post-colonization (Supplementary Fig. S6). Histological evaluation did not reveal damage to the mucosa (Fig. 4f). The increase in paracellular permeability at day 7 post-colonization was also observed in a supplemental experiment in which mice were colonized by an alternative mode with the same donor instead of intragastric gavage, whereby the microbial inoculum was applied to the face and paws (Supplementary Fig. S7). As with SPF mice, no significant differences in SERT, ASBT, CFTR, DRA, MCT-1, NPC1L1, NHE3, or SMCT-1 expression were observed (Supplementary Fig. S8). Overall, these findings suggest that colonic paracellular permeability and some TJ protein characteristics are dynamically and rapidly affected by colonization, in a manner that resembles the steady state observed in conventional mice.

### Colonization induces maturation of colonic barrier structure

**Figure 5:**
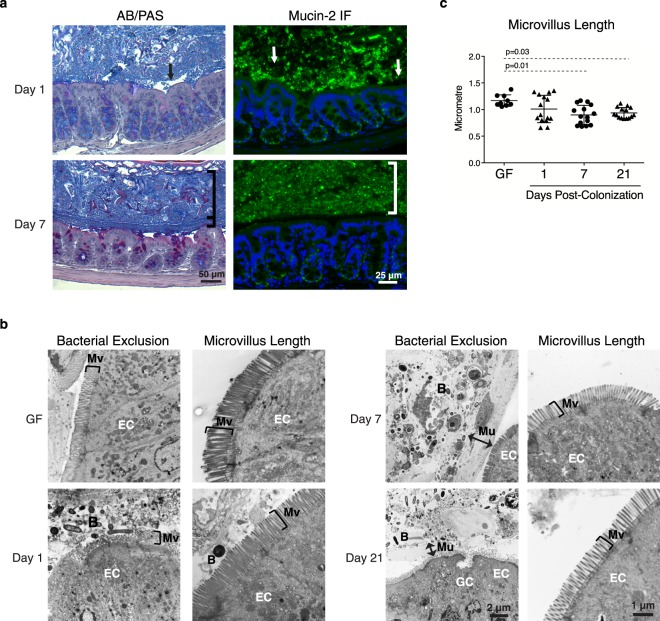
Microbiota induces maturation of colonic barrier structure. Mucus structure and microvillus length were assessed at days 1, 7 and 21 post-colonization. (**a**) Representative pictures of mucus structure evaluated by alcian blue/periodic acid-Schiff (AB/PAS) staining (left panel) and mucin-2 IF staining (right panel). Mucin-2 is shown in green, nuclei were stained with DAPI (blue). Arrows designate areas of inconsistent mucus, brackets indicate areas of uniform, thick mucus. (**b**) Representative EM images demonstrating bacterial exclusion (left images) and microvillus length (right images). Markings indicate microvilli (Mv), epithelial cells (EC), and the black arrows indicate separation of bacteria (B) from the epithelium by the mucus (Mu) layer. (**c**) Microvillus length. Each data point represents analysis of one image; the horizontal line and whiskers depict the mean ± SD. Data was collected over 2–5 independently executed experiments. Significance was determined by Kruskal-Wallis test with Dunn’s post-hoc test.

We focused our structural immunohistochemistry (IHC) and EM analysis between days 1 and 7 post-colonization, when paracellular permeability, microbiota and TJ protein expression changes were more marked. At day 1 post-colonization, a thin, patchy mucus layer composed primarily of alcian blue-stained mucins was observed, as well as direct contact between microbes and ECs (Fig. 5a,b). By day 7 post-colonization, a mixture of alcian blue- and periodic acid/Schiff-stained mucins were produced, and although 16S rRNA gene and mucin-2 staining demonstrated bacteria were present in the inner mucus layer of the proximal colon, the glycocalyx physically segregated bacteria from the epithelial surface (Fig. 5a,b; Supplemental Fig. S9). Bacterial exclusion from the epithelium was maintained up to day 21 post-colonization (Fig. 5b). A 20% reduction in microvillus length was observed by day 7 post-colonization, resembling the structure of SPF mice (Fig. 5b,c). Bacterial exclusion and microvillus shortening were also observed in mice colonized for 7 days using the aforementioned alternative mode of colonization (Supplementary Fig. S10). These results indicate mucus and microvillus structure reach the physiological state by day 7 post-colonization.

### Colonization induces a transient increase in IL-18 production by colonic epithelial cells

**Figure 6:**
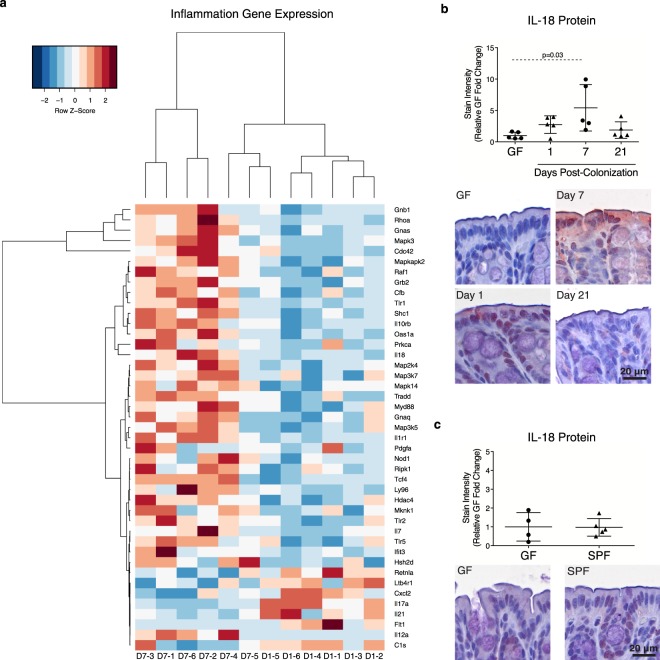
Microbiota induces a transient increase in colonic epithelial cell IL-18 at day 7 post-colonization. (**a**) Heat map of significantly altered Log2 transformed inflammation-associated genes in the colonic IEL-enriched compartment as assessed by NanoString at days 1 and 7 post-colonization (D1 and D7, respectively, followed by sample number 1–6). Evaluation of IL-18 protein expression by IHC and representative images (**b**) post-colonization and (**c**) in SPF mice. Each data point represents one mouse; the horizontal line and whiskers depict the mean ± SD. Data was collected over 2–5 independently executed experiments. Significance was determined by Kruskal-Wallis test with Dunn’s post-hoc test or an unpaired, two-tailed Student’s t test.

A number of cytokines produced by cells in the epithelial compartment, such as intraepithelial lymphocytes (IELs) and ECs, regulate intestinal permeability^15–19^. In order to gain insight into mechanisms that may influence colonic barrier changes post-colonization, expression of proinflammatory genes in the IEL-enriched compartment of the colon were evaluated. Out of a panel of 254 genes, 43 were found to be significantly different between days 1 and 7 post-colonization (Fig. 6a; Supplementary Fig. S11). No significant changes in *Tnf*, *Ifng*, *Il6*, or *Il1b* were found (Supplementary Fig. S12), but *Il18* was increased at day 7 post-colonization. IL-18 is a regulator of paracellular permeability that is produced by ECs^20–23^. Therefore, IL-18 localization and expression was assessed by IHC. At day 7 post-colonization, an increase in IL-18 protein expression was detected in surface ECs compared to GF mice (Fig. 6b). By day 21 post-colonization, the IL-18 expression level had diminished, resembling that observed in SPF mice (Fig. 6b,c). These findings suggest transient epithelial IL-18 production at day 7 post-colonization may drive physiological changes in the expression of TJ proteins and paracellular permeability.

### Barrier adaptations post-colonization reduce systemic microbial antigen exposure

**Figure 7:**
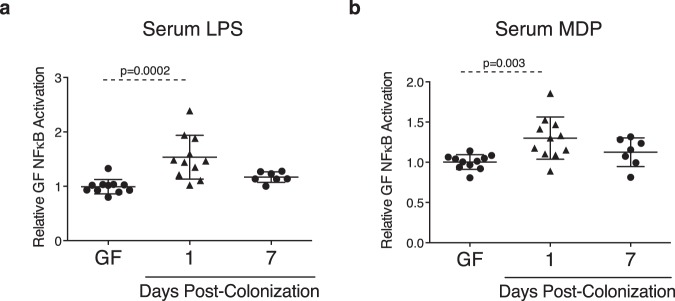
Barrier adaptations post-colonization limit systemic microbial antigen exposure. Bacterial components (**a**) LPS and (**b**) MDP were assessed in serum at days 1 and 7 post-colonization. Each data point represents one mouse; the horizontal line and whiskers depict the mean ± SD. Data was collected over 2–5 independently executed experiments. Significance was determined by Kruskal-Wallis test with Dunn’s post-hoc test.

To gain insight into the functional significance of structural and permeability changes post-colonization, immunostimulatory bacterial components lipopolysaccharide (LPS) and muramyl dipeptide (MDP) were semi-quantified in serum at days 1 and 7 post-colonization. Both LPS and MDP were increased at day 1 post-colonization, while no significant changes were observed at day 7 post-colonization (Fig. 7). These findings indicate colonization-induced barrier adaptations are associated with a transient increase in circulating bacterial products that resolves as barrier function reaches the physiological state.

### Resistance to DSS-induced intestinal inflammation increases within a week of colonization

**Figure 8:**
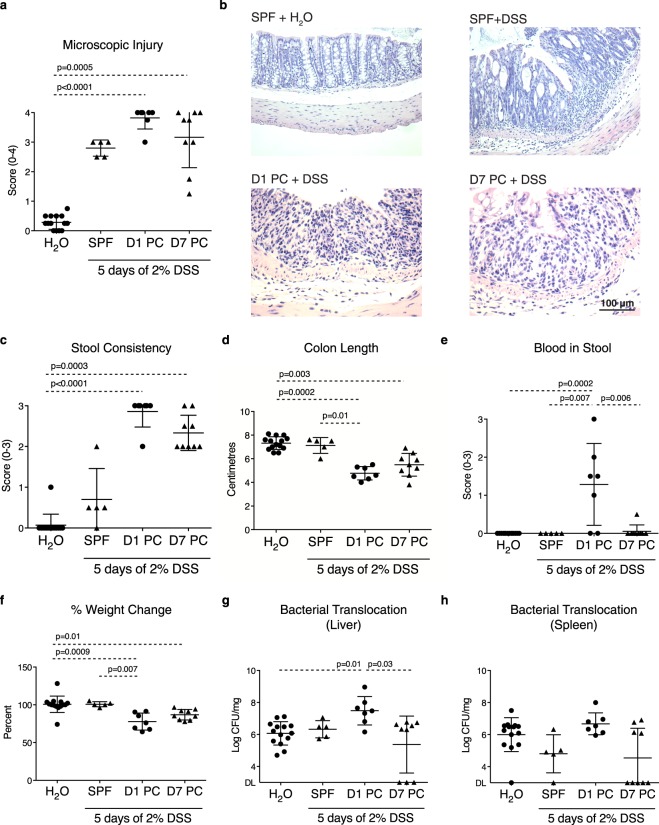
Barrier adaptations post-colonization increase resistance to DSS-induced injury. SPF mice and mice at days 1 and 7 post-colonization (PC) were given 2% DSS in drinking water for 5 days and sacrificed 2 days thereafter to assess mucosal injury, inflammation, and bacterial translocation. Mice given water were used as controls for each group and pooled together. (**a**) Microscopic injury score and (**b**) representative haematoxylin and eosin stained images. (**c**) Stool consistency score. (**d**) Colon length. (**e**) Blood in stool score. (**f**) Percent weight change. (**g**) Quantification of anaerobic bacteria in the liver and (**h**) spleen. DL = detection limit (3.33 log CFU/mg of tissue). Each data point represents one mouse; the horizontal line and whiskers depict the mean ± SD. Data was collected over 2 independently executed experiments. Significance was determined by Kruskal-Wallis test with Dunn’s post-hoc test.

To evaluate the pathophysiological significance of intestinal barrier structural and permeability changes stimulated by colonization, intestinal injury and subsequent microbiota-driven inflammation was induced using the DSS model. SPF mice and mice colonized for 1 and 7 days were exposed to low dose (2%) DSS for 5 days. SPF mice developed moderate microscopic damage (Fig. 8a,b). Compared to SPF mice, mice colonized for 1 day prior to DSS administration had significantly higher microscopic damage and weight loss, severe diarrhea, colon shortening, blood detectable in stool, translocation of live bacteria to the liver, as well as a non-statistically significant trend of translocation to the spleen (Fig. 8a–j). Mice colonized for 7 days prior to DSS exhibited diarrhea and colon shortening, but weight loss was less marked, and no significant bacterial translocation was observed. Fluid consumption was monitored throughout the experiment, and mice colonized for 7 days consumed significantly more DSS than SPF and 1-day colonized mice (average DSS/water consumption: 19 ± 0.93 ml SPF mice, 28 ± 3.9 ml 1-day colonized, 40 ± 4.4 ml 7-day colonized (p < 0.0001 vs SPF), 25 ± 5.9 ml water control (mean ± SD; n = 5–14)). These findings suggest a longer colonization period is associated with higher resistance to DSS-induced inflammation.

## Discussion

Commensal colonization induces mucosal and systemic immune maturation important for the maintenance of homeostasis^1–4,24,25^; however, the role of microbiota in shaping intestinal barrier structure and permeability is poorly defined, and most studies have been performed using selected commensals or murine microbiota. Here we determined that within a week of colonization with a complex human gut-derived microbiota, dynamic changes in several aspects of the colonic barrier occur that favor development of a steady state between the microbiota and host.

We first determined that, compared to conventional SPF mice, GF mice had lower paracellular uptake of an inert probe in the proximal colon, suggesting the microbiota is necessary for establishment of some semi-permeable paracellular features of the colon. To study the dynamics of how these barrier changes are established, adult GF mice were then colonized with fecal microbiota from one healthy human donor extensively screened for the absence of common pathogens^14^. In agreement with previous findings, the fecal bacterial taxonomic composition of colonized mice changed significantly over the first week of colonization but was less marked between days 7 and 21 post-colonization, although higher interindividual variation in lower abundant taxa was observed at day 21 post-colonization^3,6,26^. Paracellular permeability of the colon reached the physiological state within a week of colonization. This was not associated with mucosal damage, and was independent of the colonization mode, suggesting it was not due to an overt inflammatory response towards the microbial inoculum or the intragastric gavage procedure. Furthermore, elevated systemic exposure to bacterial immunostimulatory components occurred prior to paracellular permeability reaching the physiological state, which we believe may be attributable to increased bacterial exposure when mucus mucus integrity is low, allowing passage through the tight junction “leak” pathway^27^. Paracellular permeability changes post-colonization were paralleled by decreased intercellular claudin-1 expression and a transient increase in epithelial IL-18. Although previous studies have demonstrated different microbiota shifts and changes in other cytokines following colonization, differences in study design and methodologies, including the use of murine versus human-derived microbiota, may account for discrepant profiles induced^3,15,28^. In support of our findings in this colonization model, IL-18 has previously been associated with changes in claudin-1 expression and increased paracellular permeability^29^. IL-18 has been postulated to play a dichotomous role in barrier homeostasis depending on the cellular source and stage of inflammation; on one hand, epithelial IL-18 promotes barrier integrity during initiation of inflammation, while immune cell-derived IL-18 enhances inflammation at later stages^19,20,22,23,30–32^. The modest, transient increase in epithelial IL-18 observed in this study occurred in the absence of tissue damage or inflammation, consistent with a homeostatic response. Furthermore, increased IL-18 was concurrent with lower systemic exposure to bacterial immunostimulatory components, as well as decreased bacterial translocation and susceptibility to inflammation following DSS-induced injury. Together, these findings indicate that commensal colonization stimulates induction of physiological paracellular permeability within a week, in association with transient IL-18 production and diminished claudin-1 expression, without compromising overall barrier integrity.

Structural integrity of the colonic barrier is essential for limiting host-microbe interactions and maintaining proper barrier function. Our results show that the structure of the proximal colon mucus layer, comparable to that observed in SPF mice, was induced within the first week of colonization. This led to segregation of bacteria from the epithelial surface by the glycocalyx and coincided with reduced systemic microbial antigen exposure. In support of this, fortification of the mucus layer and increased diversity of mucin glycosylation was observed within 48 hours of human intestinal organoid colonization with human-derived, non-pathogenic *E. coli*^33^. Johansson *et. al*.^34^ demonstrated exclusion of bacteria-sized beads by the mucus layer at 2 weeks post-colonization of GF mice with conventional murine cecal microbiota, although the area of exclusion was smaller than that of conventional mice and was not observed in all samples. In contrast to our EM findings, fluorescence *in situ* hybridization of bacterial DNA also suggested bacteria were in contact with colonic epithelium at 2 weeks post-colonization^34^. It is possible differing techniques of assessment and microbiota background account for some of these differences, as host and microbiota-specific effects on mucus structure have been described^10,35,36^. Nevertheless, our finding that mucus fortification is associated with increased resistance against intestinal injury and bacterial translocation is in accordance with previous studies^37–41^. Overall, our data demonstrates mucus fortification occurs rapidly following colonization, coinciding with TJ protein expression changes and physiological paracellular permeability induction, which may play a role in controlling microbial antigen uptake.

In summary, features of the intestinal epithelial colonic barrier characteristic of conventional mice were reached within a week of commensal human microbiota colonization. This coincided with shifts in bacterial taxonomic structure and decreased claudin-1 expression, as well as transiently increased epithelial IL-18. Since colonization was performed beyond the postnatal and weaning periods to avoid age-related developmental and diet confounders, the results indicate microbiota-mediated effects on certain characteristics of colonic barrier maturation are not restricted to a window of perinatal exposure, as has been reported for aspects of immune maturation^42,43^. Such findings support the belief that the microbiota continually affects barrier function and integrity throughout life, and may contribute to development of barrier dysfunction and chronic intestinal disorders in adulthood^44,45^. Additionally, we also determined post-colonization changes resulted in a more resilient barrier capable of limiting systemic microbial exposure and increasing resistance to chemically induced intestinal injury. We believe this constitutes a useful and practical model to investigate the therapeutic potential of barrier-modulating microbiota therapies and the role of disease-associated microbial communities, or individual strains, on intestinal barrier dysfunction.

## Methods

### Mice

Male and female adult GF C57BL/6 mice (2–3 months old) on S-2335 Mouse Breeder Sterilizable Diet (Teklad) were obtained from the Axenic Gnotobiotic Unit at McMaster University. Conventionally raised, adult, male and female SPF C57BL/6 mice raised on NIH #31 M Rodent Diet were obtained from Taconic (Taconic Biosciences Inc, Germantown, New York; from rooms IBU04 and IBU14; 2–3 months old) and used for experiments after a week of acclimatization to the McMaster Animal Facility and S-2335 Mouse Breeder Sterilizable Diet (Teklad). Animals were kept on a twelve-hour light and dark cycle, and housed in sterile vent/rack cages with *ad libitum* access to food (Teklad S-2335 Mouse Breeder Sterilizable Diet) and water at the McMaster Animal Facility. All experiments were performed in accordance with McMaster University animal utilization protocols, with approval from the McMaster University Animal Care Committee, and conducted under the Canadian Council on Animal Care Guidelines.

### Overall design

Comparisons between GF and SPF mice as well longitudinal studies in colonized mice at days 1, 7 and 21 post-colonization were performed. For the latter, GF mice were colonized by intragastric gavage with 0.2 ml of fecal slurry (1:10 w/v) from one healthy adult human donor previously shown to be free of known pathogens (Supplementary Fig. S3)^14^. Fecal slurry was prepared as described previously^44^. In a supplemental experiment, fecal slurry from the same donor was applied to the face and front paws of GF mice instead of by intragastric gavage, as an alternative colonization mode. Independent experiments describe individual experimental replicates in which samples were obtained from mice colonized and/or sacrificed together on the same day.

### Intestinal permeability measurements

Intestinal permeability was evaluated *ex vivo* using Ussing chambers as previously described^46^. Paracellular and transcellular permeability were assessed using 6μCi/ml of ^51^Cr-EDTA and 5 × 10^5^ M HRP (type II; Sigma Aldrich) probes, respectively. Serosal ^51^Cr-EDTA was quantified using a liquid scintillation counter and reported as percent recovery/cm^2^/hour. A modified Worthington assay was used to quantify serosal HRP^47^.

### RNA extraction

Fresh colon tissue or the IEL-enriched compartment were incubated overnight at 4 °C in RNAlater (Life Technologies). Colon tissues were stored at −20 °C. RNA was extracted using the RNeasy Mini Kit (Qiagen) with DNase treatment. RNA concentration was determined using a NanoDrop spectrophotometer (Qiagen) and quality assessed by agarose gel electrophoresis.

### Real-time quantitative PCR of tight junction and apical barrier transport proteins

To evaluate tight junction protein gene expression, reverse transcription of 1 μg of RNA template was performed using iScript Reverse Transcriptase (Bio-Rad). Quantitative real-time PCR was performed using cDNA (50 ng/μl), primers (0.5 μM) and SsoFast EvaGreen Supermix (Bio-Rad), (Supplementary Table S1), and amplified using a Mastercycler ep realplex^4^ (Eppendorf). Enzyme activation was induced at 95 °C for 30 seconds, denaturation at 95 °C for 5 seconds and annealing/extension at 60 °C for 20 seconds was cycled 45 times.

For assessment of apical membrane transport protein gene expression, 50 ng of cDNA with 0.15 μM primers (Supplementary Table S1) was reverse transcribed and amplified in one step reaction using Brillliant III Ultra-Fast SYBR Green qPCR Master Mix (Agilent Technologies) in Stratagene Mx3005P (Agilent Technologies). PCR proceeded 40 cycles of denaturation at 95 °C for 20 seconds and annealing/extension at 60 °C for 22 seconds. Gene expression of tight junction proteins and apical membrane transport proteins was normalized to *Gapdh*, and the fold expression changes relative to the GF mouse *Gapdh* mean determined using the 2^−ΔΔCT^ method.

### Mucin-2 and TJ protein expression by immunofluorescence

Mucin-2 and TJ protein expression in the proximal colon were assessed by immunofluorescent staining of formalin-fixed, paraffin-embedded sections. Sections were cut to 4 μm, deparaffinized and rehydrated. For claudin-1 antigen retrieval, sections were steamed in sodium citrate buffer (pH 6.0) for 30 minutes. For occludin antigen retrieval, sections were subjected to proteinase K (20 μg/ml) for 10 minutes at room temperature. For claudin-1 and occludin staining, tissue sections were blocked with 2% BSA/PBS/0.05% Tween 20 for 1 hour. For ZO-1 staining, sections were treated 0.4% pepsin in 0.01 N HCl for 30 minutes at 37 °C, and blocked with 10% goat serum for 1 hour at room temperature. Tissue sections were incubated overnight at 4 °C with one of the following rabbit primary antibodies in 1% BSA/PBS/0.05% Tween 20: claudin-1 (1:100; Abcam ab15098), occludin (1:200; Bioss Antibodies bs-1495R), or ZO-1 (1:200; Invitrogen 40–2200). Slides were incubated with goat anti-rabbit Alexa Fluor 488 (1:250; Thermo Fisher Scientific A11070) secondary antibody for 2 hours at room temperature, and mounted with Prolong Gold Antifade with DAPI (Thermo Fisher Scientific). Controls included slides stained in the absence of primary antibody as well as absorptive controls, where primary antibody was applied with excess peptide. Carnoy’s-fixed (60% methanol, 30% chloroform, 10% glacial acetic acid), paraffin-embedded slides were used for mucin-2 (1:100; Abcam ab76774) staining, as well as with the aforementioned secondary antibody. Images were acquired using ImagePro Plus (Media Cybernetics). Staining was blindly assessed using ImageJ v.1.49 (NIH), and only samples with appropriate tissue orientation and sectioning were evaluated. Semi-quantification of protein expression is reported as the fold increase of signal intensity relative to the GF controls, which was arbitrarily set to 1.

### Evaluation of intestinal structure by EM

Cross sections of colon no more than 0.4 cm thick were fixed overnight with 2% glutaraldehyde (v/v) in 0.1 M sodium cacodylate buffer (pH 7.4) at 4 °C. Sections were post-fixed with 1% osmium tetroxide in 0.1 M sodium cacodylate buffer, dehydrated, rinsed with propylene oxide, subjected to a graded series of Spurr’s resin, polymerized overnight in embedding moulds filled with 100% Spurr’s resin at 60 °C, cut using a Leica UCT ultramicrotome, and picked up onto Cu grids. Sections were post-stained with uranyl acetate and lead citrate, viewed using a JOEL JEM 1200 EX TEMSCAN transmission electron microscope at an accelerating voltage of 80 kV. Images were obtained using an AMT 4-megapixel digital camera (Advanced Microscopy Techniques).

EM images with clear microvillus cross sectioning were used to measure microvillus length in ImageJ (NIH). Colon sections from three mice were evaluated per group, 5–6 images evaluated per section, and 10 longitudinally cut microvilli measured per image. Each data point depicts the average microvillus length (μm) per image.

### Microbiota analysis

Fecal samples from the same 7 mice were collected at days 1, 7 and 21 post-colonization, flash frozen, and stored at −80 °C. DNA was extracted using the MagMAX Express 96 (Life Technologies), the hypervariable V3 region of the bacterial 16S rRNA gene was sequenced using the Illumina MiSeq platform, and analysis was performed as described previously^48,49^. Briefly, sequences were edited using Cutadapt version 1.2.1^50^, aligned using PANDAseq v.2.8^51^, Abundant OTU was used to select operational taxonomic units^52^, Greengenes reference database was used to assign taxonomy, and QIIME was used to filter out operational taxonomic units below 0.01% abundance^53^. The fecal microbiota was sampled at a mean depth of 98 779 reads per sample (median = 83 515). Operational taxonomic unit abundance and Firmicutes:Bacteroidetes graphs were created in GraphPad Prism 6.0 (GraphPad Software, Inc). Principal component analysis using unweighted UniFrac and Bray Curtis dissimilarity ordinations was performed in QIIME.

### Evaluation of mucus layer structure and bacterial distribution by staining

Proximal colon mucus layer structure was assessed using alcian blue and periodic acid Schiff staining of Carnoy’s-fixed (60% methanol, 30% chloroform, 10% glacial acetic acid), paraffin-embedded sections. Deparaffinized, rehydrated tissue sections were treated with 3% acetic acid for 3 minutes, alcian blue for 15 minutes, periodic acid for 5 minutes, Schiff’s reagent for 15 minutes, and counterstained with haematoxylin.

Distribution of bacteria in the mucus of the proximal colon was evaluated by 16S rRNA gene fluorescence *in situ* hybridization and mucin-2 IF staining of Carnoy’s-fixed, paraffin-embedded sections. Deparaffinized, rehydrated tissue sections were hybridized overnight with 5 ng/μl of 16S rRNA gene probe EUB338 (5′-5Cy3 GCT GCC TCC CGT AGG AGT-3′) at 50 °C in 20 mM Tris-HCl (pH 7.4), 0.9 M NaCl, 0.1% SDS. Sections were blocked for 30 minutes at 4 °C with 5% fetal bovine serum, and incubated overnight at 4 °C with rabbit anti-mucin-2 (1:200; Abcam ab76774) in blocking solution. Sections were incubated for 2 hours at room temperature with goat anti-rabbit Alexa Fluor 488 (1:250; Thermo Fisher Scientific A11070) secondary antibody, and mounted with Prolong Gold Antifade with DAPI (Thermo Fisher Scientific). Controls included slides stained in the absence of EUB338 probe or primary antibody. ImagePro Plus (Media Cybernetics) was used to acquire images.

### Expression of inflammation-associated genes in the IEL-enriched compartment

The IEL-enriched compartment of the colon was isolated using a modified protocol^54^. Briefly, colons were collected, cleared of contents and cut into 3–5 mm pieces. Tissue pieces were incubated in DTT/HEPES/Hank’s for 15 minutes at 37 °C on a shaker, and 5 times in EDTA/HEPES/Dulbecco’s PBS, with vortexing and straining between incubations. Supernatant was passed through 70 μm strainers and the IEL-enriched compartment isolated by Percoll gradient. Cells were resuspended in RNAlater and kept at 4 °C overnight prior to RNA extraction. Quality of RNA was first checked using an RNA Nano Chip and Agilent 2100 Bioanalyzer (Agilent Technologies). Gene expression was determined using the nCounter^®^ Mouse Inflammation v2 XT CodeSet (NanoString Technologies Inc; Supplementary Table S2), analysed with nSolver Analysis Software v2.5.34 (NanoString Technologies Inc), and normalized Log2 transformed gene values depicted in heat maps with Euclidean clustering created in RStudio v1.0.143 (RStudio Inc).

### IL-18 expression by immunohistochemistry

Formalin-fixed, paraffin-embedded colon sections were cut to 4 μm, deparaffinized, rehydrated, and blocked with dual endogenous enzyme blocking reagent (Dako S2003) for 10 minutes. Slides were steamed in sodium citrate buffer (pH 6.0) for 30 minutes, blocked with 2% BSA/PBS/0.05% Tween 20 for 2 hours, as well as Peroxidase Block (Dako K4004) for 5 minutes. Samples were stained with rabbit anti-mouse IL-18 (1:100; Abcam ab71495) overnight at 4 °C in 1% BSA/PBS/0.05% Tween 20, treated with Envision System-HRP Labeled Polymer Anti-Rabbit (Dako K4003) for 30 minutes, AEC substrate chromogen (Dako K3464), counterstained with haematoxylin and mounted with Aqua-Mount (Thermo Fisher Scientific). Controls included slides stained in the absence of primary antibody as well as an absorptive control, where primary antibody was applied with excess peptide. Images were acquired using ImagePro Plus (Media Cybernetics). Staining was blindly assessed using the IHC Toolbox in ImageJ (NIH) and shown as the fold increase of signal intensity relative to the GF controls, which was arbitrarily set to 1.

### TLR4 and NOD2 NFκB-SEAP reporter assays

To detect LPS and MDP in serum, colorimetric NFκB-SEAP reporter assays were performed as described previously^55^. Briefly, heat inactivated serum diluted 1:5 in PBS, 1:1 in water, and finally 10 ul into 190 ul in HEK Blue Detection Media (Invitrogen) was incubated with HEK293 cell line expressing TLR4, MD2 and CD14 transfected with pNifty2-SEAP for LPS detection, or HEK293T cells transfected with NOD2 and pNifty2-SEAP for MDP detection. After a 24-hour incubation at 37 °C, readings were performed at 630 nm, and NFκB activation relative to the GF controls calculated.

### Dextran sodium sulfate-induced intestinal injury

Intestinal injury and inflammation were induced by administration of 2% DSS (36,000–50,000 MW; MP Biomedicals LLC) in drinking water for 5 days, followed by 2 days on normal drinking water prior to sacrifice. Negative control mice for each group were given normal drinking water, and were pooled into one group as no significant differences were observed. Colon length, percent weight change, stool consistency and presence of blood were evaluated at sacrifice. Stool consistency and blood were each scored on a scale of 0–3 as outlined in Supplementary Table S3. Haematoxylin and eosin stained colon sections were blindly evaluated for microscopic damage on a scale of 0–4 as previously described^56^.

### Bacterial translocation

Translocation of live bacteria was evaluated by homogenizing liver and spleen (1:10 w/v) in pre-reduced PBS, plating duplicate serial dilutions on brain heart infusion media (BD) supplemented with 0.5% sheep’s blood (Cedarlane), and incubating at 37 °C under anaerobic conditions for 48 hours. Data is shown as the log CFU/mg of tissue.

### Statistics

Significance was determined by Kruskal-Wallis test with Dunn’s post-hoc test or an unpaired, two-tailed Mann-Whitney test, when appropriate. For microbiota analysis, Kruskal-Wallis tests with Bonferroni correction were performed in QIIME. *P* < 0.05 was considered significant.

## Electronic supplementary material

Supplementary Information

## Data Availability

The 16S gene sequencing dataset generated during this study is available in NCBI’s SRA repository with BioProject ID PRJNA430097; http://www.ncbi.nlm.nih.gov/bioproject/430097.
