# Supplementary material for: Commensal microbiota induces colonic barrier structure and functions that contribute to homeostasis
Source: Sci Rep. 2018 Sep 21;8:14184. doi: 10.1038/s41598-018-32366-6 (PMC6155058; doi:10.1038/s41598-018-32366-6)
Supplement: Supplementary file 1 — Supplementary Information [file 41598_2018_32366_MOESM1_ESM.pdf]

SUPPLEMENTARY MATERIAL

Commensal microbiota induces colonic barrier structure and functions that contribute to homeostasis.

Christina L. Hayes[1], Heather J. Galipeau[1], Jasmine Dong[1], Jennifer Jury[1], Justin McCarville[1], Xianxi Huang[1], Xuan-Yu Wang[1], Avee Naidoo[2,3], Arivarasu N. Anbazhagan[4], Josie Libertucci[1], Conor Sheridan[1], Pradeep K. Dudeja[4,5], Dawn M. E. Bowdish[2,3], Michael G. Surette[1,6], and Elena F. Verdu[1*].

SUPPLEMENTARY TABLES

Supplemental Table S1. RT-qPCR primers.

Supplementary Table S2. Inflammation-associated genes in the nCounter® Mouse Inflammation v2 XT NanoString CodeSet.

Supplemental Table S3. Scoring systems for stool consistency and presence of blood.

SUPPLEMENTARY FIGURES

Supplementary Figure S1. Tight junction gene and protein expression in germ-free compared to conventional mice.

Supplementary Figure S2. Colonic apical membrane protein transporter expression.

Supplementary Figure S3. Donor microbiota taxonomic composition and structure.

Supplementary Figure S4. Bray Curtis dissimilarity post-colonization.

Supplementary Figure S5. Alpha diversity is not significantly changed post-colonization.

Supplementary Figure S6. Tight junction gene and protein expression post-colonization.

Supplementary Figure S7. Paracellular permeability changes by day 7 post-colonization are independent of colonization mode.

Supplementary Figure S8. Colonic mRNA expression of apical membrane protein transporters are not significantly changed post-colonization.

Supplementary Figure S9. Bacterial distribution in the mucus layer of the proximal colon.

Supplementary Figure S10. Induction of colon structural changes by day 7 post-colonization is independent of colonization mode.

Supplementary Figure S11. Heat map of inflammation-associated gene expression in the colonic IEL-enriched compartment.

Supplementary Figure S12. Expression of cytokine genes in the colonic IEL-enriched compartment at days 1 and 7 post-colonization.

Supplemental Table S1. RT-qPCR primers.

	Protein	Gene	Forward	Reverse
Apical Junction Proteins	Claudin-1	*Cldn1*	5’-GGACTGTGGATGTC CTGCGTTT-3’	5’-GCCAATTACCATCAA GGCTCGG-3’
	Claudin-3	*Cldn3*	5’-TCATCGTGGTGTCC ATCCTGCT-3’	5’AGAGCCGCCAACAGG AAAAGCA-3’
	Occludin	*Ocln*	5’-TGGCAAGCGATCAT ACCCAGAG-3’	5’-CTGCCTGAAGTCATC CACACTC-3’
	ZO-1	*Tjp1*	5’-GTTGGTACGGTGCC CTGAAAGA-3’	5’GCTGACAGGTAGGAC AGACGAT-3’
	GAPDH	*Gapdh*	5’-CCATGGAGAAGGC TGGGG-3’	5’-CAAAGTTGTCATGGA TGACC-3’
Protein Transporters	ASBT	*Slc10a2*	5’-TGGGTTTCTTCCTG GCTAGACT-3’	5’-TGTTCTGCATTCCAG TTTCCAA-3’
	CFTR	*Cftr*	5’-CTGGACCACACCA ATTTTGAGG-3’	5’-GCGTGGATAAGCTG GGGAT-3’
	DRA	*Slc26a3*	5’-TGGTGGGAGTTGTC GTTACA-3’	5’-CCCAGGAGCAACTG AATGAT-3’
	MCT-1	*Slc16a1*	5’-TTGGACCCCAGAG GTTCTCC-3’	5’-AGGCGGCCTAAAAG TGGTG-3’
	NPC1L1	*Npc1l1*	5’-TGGACTGGAAGGA CCATTTCC-3’	5’-GACAGGTGCCCCGT AGTCA-3’
	NHE-3	*Slc9a3*	5’-GGCCTTCATTCGCT CCCCAAG-3’	5’-ATGCTTGTACTCCTG CCGAGG-3’
	SMCT-1	*Slc5a8*	5’-TGCCATTTCCTTAT GGGTAGG-3’	5’-AGTGGAGTCCTTTCC GCATTA-3’
	SERT	*Slc6a4*	5’-GGCTGAGATGAGG AACGAAG-3’	5’-CTGCAAACGTGCTAT CCAAA-3’
	GAPDH	*Gapdh*	5’- TGTGTCCGTCGTGG ATCTGA-3’	5’-CCTGCTTCACCACCT CTTGAT-3’

Supplementary Table S2. Inflammation-associated genes in the nCounter® Mouse Inflammation v2 XT NanoString CodeSet.

Gene Name	Accession #	Gene Name	Accession #
Ager	NM_007425.2	Ccr3	NM_009914.4
Alox12	NM_007440.4	Ccr4	NM_009916.2
Alox15	NM_009660.3	Ccr7	NM_007719.2
Alox5	NM_009662.2	Cd163	NM_053094.2
Areg	NM_009704.3	Cd4	NM_013488.2
Arg1	NM_007482.3	Cd40	NM_011611.2
Atf2	NM_001025093.1	Cd40lg	NM_011616.2
Bcl2l1	NM_009743.4	Cd55	NM_010016.2
Bcl6	NM_009744.3	Cd86	NM_019388.3
Birc2	NM_007465.2	Cdc42	NM_009861.1
C1qa	NM_007572.2	Cebpb	NM_009883.3
C1qb	NM_009777.2	Cfb	NM_008198.2
C1ra	NM_023143.3	Cfd	NM_013459.1
C1s	NM_144938.2	Cfl1	NM_007687.5
C2	NM_013484.2	Chi3l3	NM_009892.1
C3	NM_009778.2	Creb1	NM_133828.2
C3ar1	NM_009779.2	Crp	NM_007768.4
C4a	NM_011413.2	Csf1	NM_001113530.1
C6	NM_016704.2	Csf2	NM_009969.4
C7	XM_356827.6	Csf3	NM_009971.1
C8a	NM_146148.1	Cxcl1	NM_008176.1
C8b	NM_133882.2	Cxcl10	NM_021274.1
C9	NM_013485.1	Cxcl2	NM_009140.2
Ccl11	NM_011330.3	Cxcl3	NM_203320.2
Ccl17	NM_011332.2	Cxcl5	NM_009141.2
Ccl19	NM_011888.2	Cxcl9	NM_008599.2
Ccl2	NM_011333.3	Cxcr1	NM_178241.4
Ccl20	NM_016960.1	Cxcr2	NM_009909.3
Ccl21a	NM_011124.4	Cxcr4	NM_009911.3
Ccl22	NM_009137.2	Cysltr1	NM_021476.4
Ccl24	NM_019577.4	Cysltr2	NM_001162412.1
Ccl3	NM_011337.1	Daxx	NM_007829.3
Ccl4	NM_013652.1	Ddit3	NM_007837.3
Ccl5	NM_013653.1	Defa-rs1	NM_007844.2
Ccl7	NM_013654.2	Elk1	NM_007922.4
Ccl8	NM_021443.2	Fasl	NM_010177.3
Ccr1	NM_009912.4	Flt1	NM_010228.3
Ccr2	NM_009915.2	Fos	NM_010234.2

Supplementary Table S2 Continued.

Gene Name	Accession #	Gene Name	Accession #
Fxyd2	NM_052823.2	Il1a	NM_010554.4
Gnaq	NM_008139.5	Il1b	NM_008361.3
Gnas	NM_010309.3	Il1r1	NM_001123382.1
Gnb1	NM_008142.3	Il1rap	NM_008364.2
Gngt1	NM_010314.2	Il1rn	NM_031167.4
Gpr44	NM_009962.2	Il2	NM_008366.3
Grb2	NM_008163.3	Il21	NM_021782.2
H2-Ea-ps	NM_010381.2	Il22	NM_016971.1
H2-Eb1	NM_010382.2	Il22ra2	NM_178258.5
Hc	NM_010406.1	Il23a	NM_031252.1
Hdac4	NM_207225.1	Il23r	NM_144548.1
Hif1a	NM_010431.2	Il3	NM_010556.4
Hmgb1	NM_010439.3	Il4	NM_021283.1
Hmgb2	NM_008252.3	Il5	NM_010558.1
Hmgn1	NM_008251.3	Il6	NM_031168.1
Hras1	NM_008284.2	Il6ra	NM_010559.2
Hsh2d	NM_197944.1	Il7	NM_008371.2
Hspb1	NM_013560.2	Il9	NM_008373.1
Hspb2	NM_024441.3	Irf1	NM_008390.1
Ifi27l2a	NM_029803.1	Irf3	NM_016849.3
Ifi44	NM_133871.2	Irf5	NM_012057.3
Ifit1	NM_008331.2	Irf7	NM_016850.2
Ifit2	NM_008332.2	Itgb2	NM_008404.4
Ifit3	NM_010501.1	Jun	NM_010591.2
Ifna1	NM_010502.2	Keap1	NM_016679.4
Ifnb1	NM_010510.1	Kng1	NM_023125.3
Ifng	NM_008337.1	Limk1	NM_010717.2
Iigp1	NM_021792.3	Lta	NM_010735.1
Il10	NM_010548.1	Ltb	NM_008518.2
Il10rb	NM_008349.5	Ltb4r1	NM_008519.2
Il11	NM_008350.2	Ltb4r2	NM_020490.2
Il12a	NM_008351.1	Ly96	NM_016923.1
Il12b	NM_008352.1	Maff	NM_010755.3
Il13	NM_008355.2	Mafg	XM_001002362.1
Il15	NM_008357.1	Mafk	NM_010757.2
Il17a	NM_010552.3	Map2k1	NM_008927.3
Il18	NM_008360.1	Map2k4	NM_009157.4
Il18rap	NM_010553.2	Map2k6	NM_011943.2

Supplementary Table S2 Continued.

Gene Name	Accession #	Gene Name	Accession #
Map3k1	NM_011945.2	Oasl1	NM_145209.2
Map3k5	NM_008580.4	Pdgfa	NM_008808.3
Map3k7	NM_172688.2	Pik3c2g	NM_011084.2
Map3k9	NM_177395.4	Pla2g4a	NM_008869.2
Mapk1	NM_001038663.1	Plcb1	NM_019677.1
Mapk14	NM_011951.2	Ppp1r12b	NM_001081307.1
Mapk3	NM_011952.2	Prkca	NM_011101.3
Mapk8	NM_016700.3	Prkcb	NM_008855.2
Mapkapk2	NM_008551.1	Ptger1	NM_013641.2
Mapkapk5	XM_990515.1	Ptger2	NM_008964.4
Masp1	NM_008555.2	Ptger3	NM_011196.2
Masp2	NM_010767.3	Ptger4	NM_008965.1
Max	NM_008558.1	Ptgfr	NM_008966.3
Mbl2	NM_010776.1	Ptgir	NM_008967.3
Mef2a	XM_976032.1	Ptgs1	NM_008969.3
Mef2b	NM_001045484.1	Ptgs2	NM_011198.3
Mef2c_Mm	NM_025282.2	Ptk2	NM_007982.2
Mef2d	NM_133665.3	Rac1	NM_009007.2
Mknk1	NM_021461.4	Raf1	NM_029780.3
Mmp3	NM_010809.1	Rapgef2	NM_001099624.2
Mmp9	NM_013599.2	Rela	NM_009045.4
Mrc1	NM_008625.1	Relb	NM_009046.2
Mx1	NM_010846.1	Retnla	NM_020509.3
Mx2	NM_013606.1	Rhoa	NM_016802.4
Myc	NM_010849.4	Ripk1	NM_009068.3
Myd88	NM_010851.2	Ripk2	NM_138952.3
Myl2	NM_010861.3	Rock2	NM_009072.2
Nfatc3	NM_010901.2	Rps6ka5	NM_153587.2
Nfe2l2	NM_010902.3	Shc1	NM_011368.4
Nfkb1	NM_008689.2	Smad7	NM_001042660.1
Nlrp3	NM_145827.3	Stat1	NM_009283.3
Nod1	NM_172729.2	Stat2	NM_019963.1
Nod2	NM_145857.2	Stat3	NM_213659.2
Nos2	NM_010927.3	Tbxa2r	NM_001277265.1
Nox1	NM_172203.1	Tcf4	NM_013685.1
Nr3c1	NM_008173.3	Tgfb1	NM_011577.1
Oas1a	NM_145211.2	Tgfb2	NM_009367.1
Oas2	NM_145227.2	Tgfb3	NM_009368.2

Supplementary Table S2 Continued.

Gene Name	Accession #
Tgfbr1	NM_009370.2
Tlr1	NM_030682.1
Tlr2	NM_011905.2
Tlr3	NM_126166.2
Tlr4	NM_021297.2
Tlr5	NM_016928.2
Tlr6	NM_011604.3
Tlr7	NM_133211.3
Tlr8	NM_133212.2
Tlr9	NM_031178.2
Tnf	NM_013693.1
Tnfaip3	NM_009397.2
Tnfsf14	NM_019418.2
Tollip	NM_023764.3
Tradd	NM_001033161.2
Traf2	NM_009422.2
Trem2	NM_031254.2
Tslp	NM_021367.1
Twist2	NM_007855.2
Tyrobp	NM_011662.2
Cltc	NM_001003908.1
Gapdh	NM_008084.1
Gusb	NM_010368.1
Hprt	NM_013556.2
Pgk1	NM_008828.2
Tubb5	NM_011655.4

Supplemental Table S3. Scoring systems for stool consistency and presence of blood.

Score	Stool Consistency	Blood in Stool
0	Firm stool, distinctly formed pellets	Negative Hemoccult test
1	Soft stool, elongated pellets	No visible blood, positive Hemoccult test
2	Very soft stool, no distinct pellets	Some blood visible in stool
3	Completely liquid stool	Blood visible throughout stool

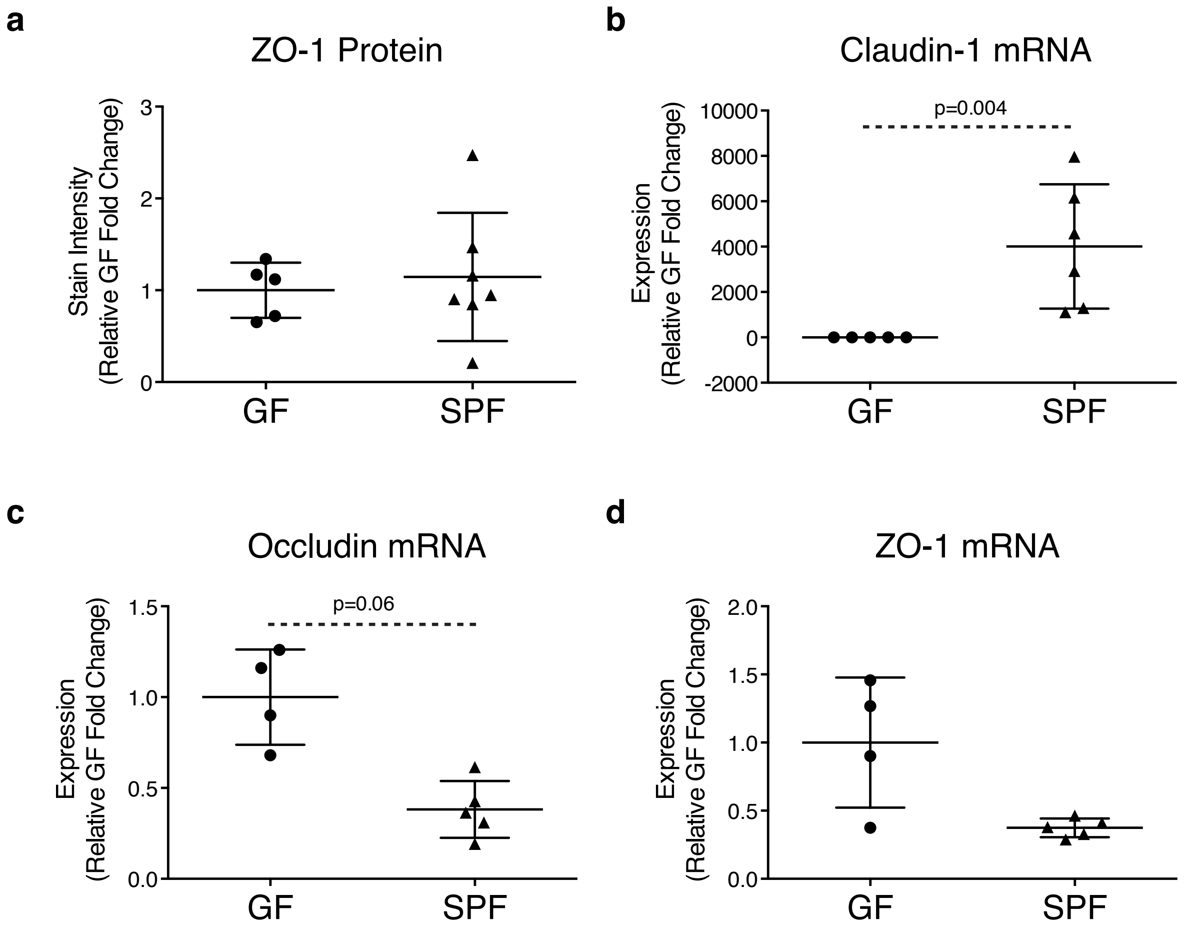

Supplementary Figure S1. Tight junction gene and protein expression in germ-free compared to conventional mice.

Tight junctions were evaluated in germ-free (GF) and conventionally raised specific pathogen free (SPF) mice. (a) Immunofluorescent staining was used to assess (d) ZO-1 protein expression. Real-time qPCR was used to evaluate mRNA expression of (b) claudin-1, (c) occludin, and (d) ZO-1 mRNA. Each data point represents one mouse; the horizontal line and whiskers depict the mean±standard deviation. Significance was assessed using by unpaired, two-tailed Mann-Whitney test.

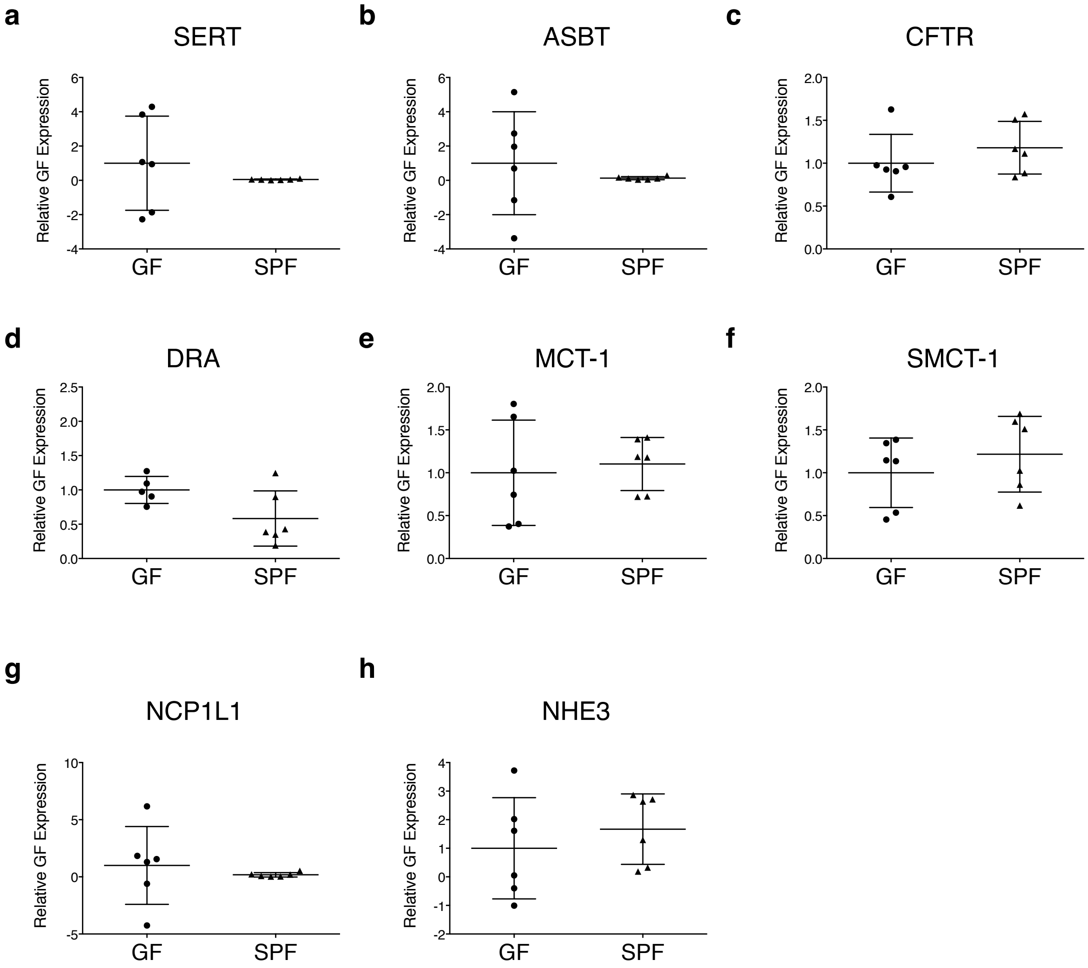

Supplementary Figure S2. Colonic apical membrane protein transporter expression. Real-time qPCR was used to evaluate colon expression of apical membrane protein transporters in germ-free (GF) and specific pathogen free (SPF) mice. (a) SERT (b) ASBT (c) CFTR (d) DRA (e) MCT-1 (f) SMCT-1 (g) NCP1L1 (h) NHE3. Each data point represents one mouse; the horizontal line and whiskers depict the mean±standard deviation. Significance was assessed by unpaired, two-tailed Mann-Whitney test.

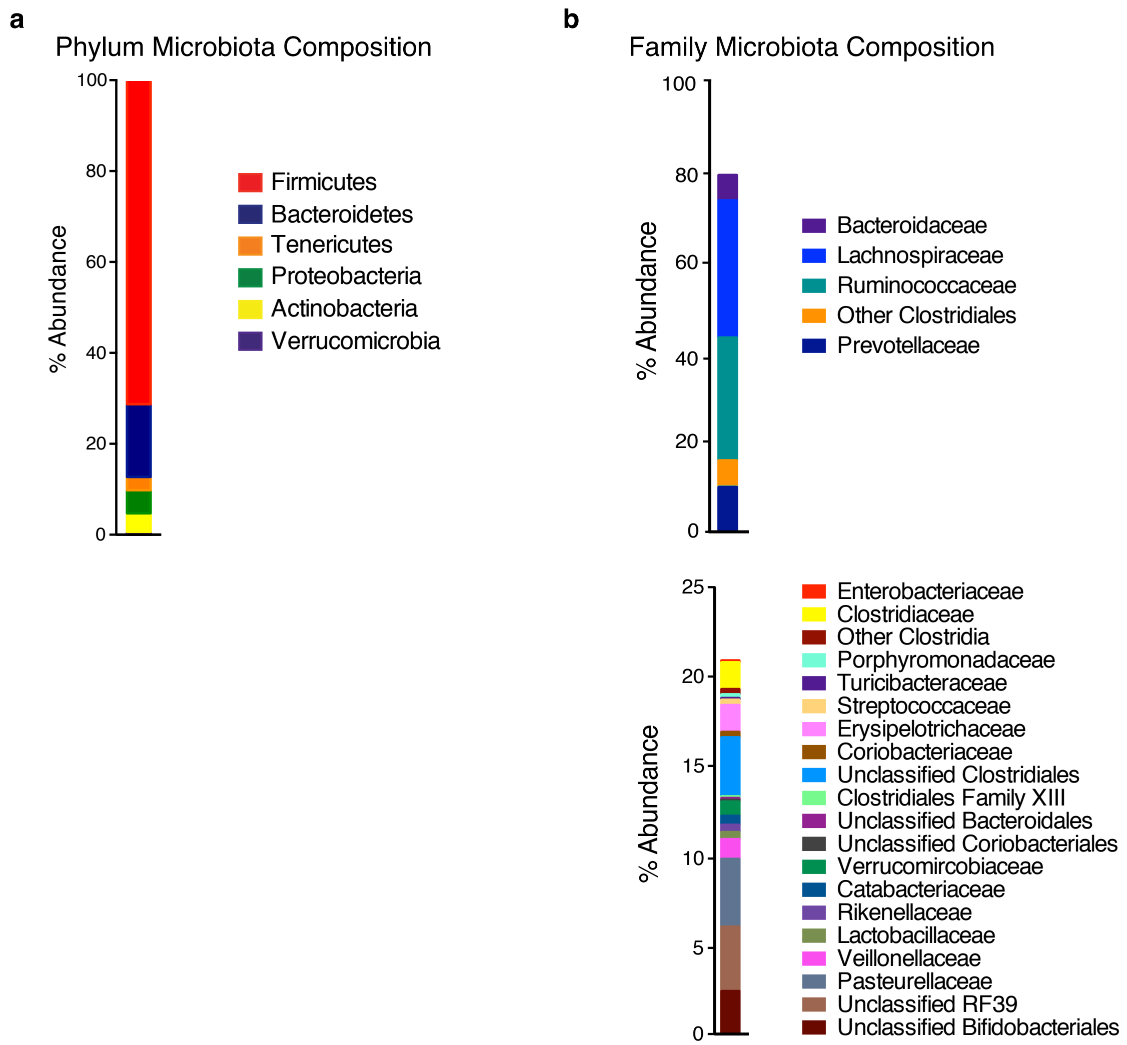

Supplementary Figure S3. Donor microbiota taxonomic composition and structure. Donor fecal microbiota inoculum was assessed by 16S rRNA gene sequencing at the (a) phylum and (b) family levels.

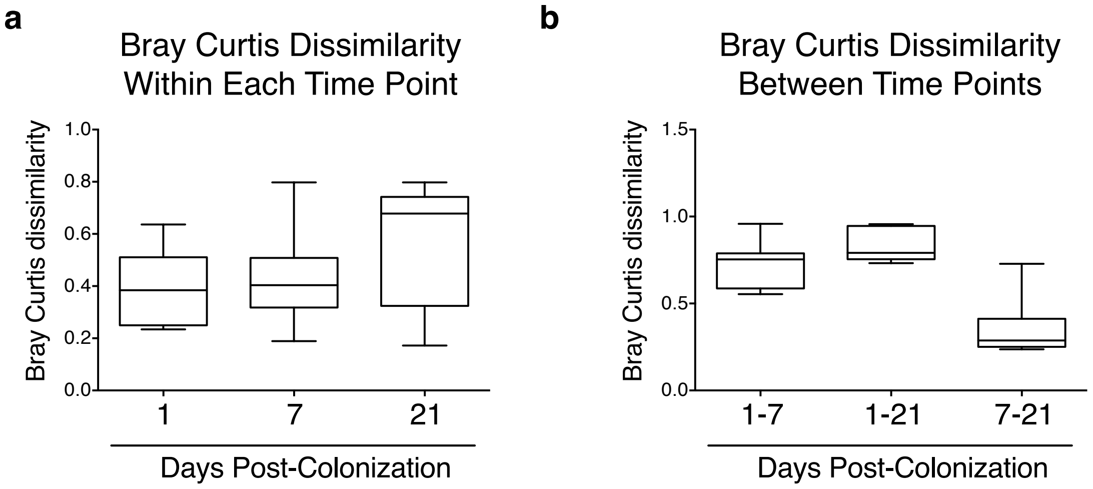

Supplementary Figure S4. Bray Curtis dissimilarity post-colonization.
16S rRNA gene sequencing was performed on fecal samples collected in a single experiment from the same mice (n=7) at days 1, 7 and 21 post-colonization with human fecal microbiota. (a) Bray Curtis dissimilarity between mice within each time point. (b) Bray Curtis dissimilarity of the individual mice between time points. Whiskers depict the minimum and maximum.

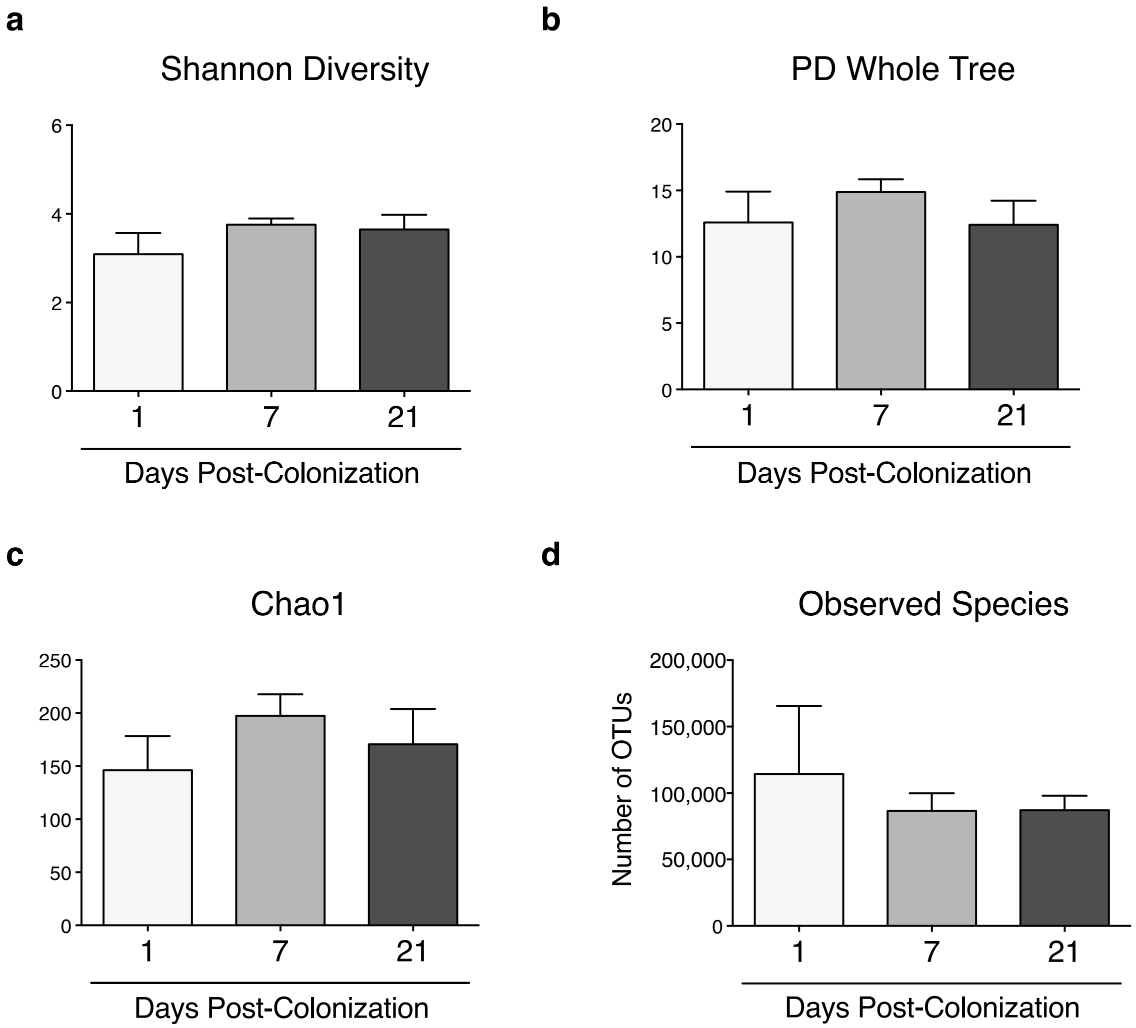

Supplementary Figure S5. Alpha diversity is not significantly changed post-colonization. 16S rRNA gene sequencing from fecal samples collected in a single experiment from the same mice (n=7) at days 1, 7 and 21 post-colonization with human fecal microbiota does not indicate significant changes in (a) Shannon diversity, (b) PD whole tree, (c) Chao1 or (d) observed species were found. Significance was evaluated using a Kruskal-Wallis test with Dunn's post-hoc test.

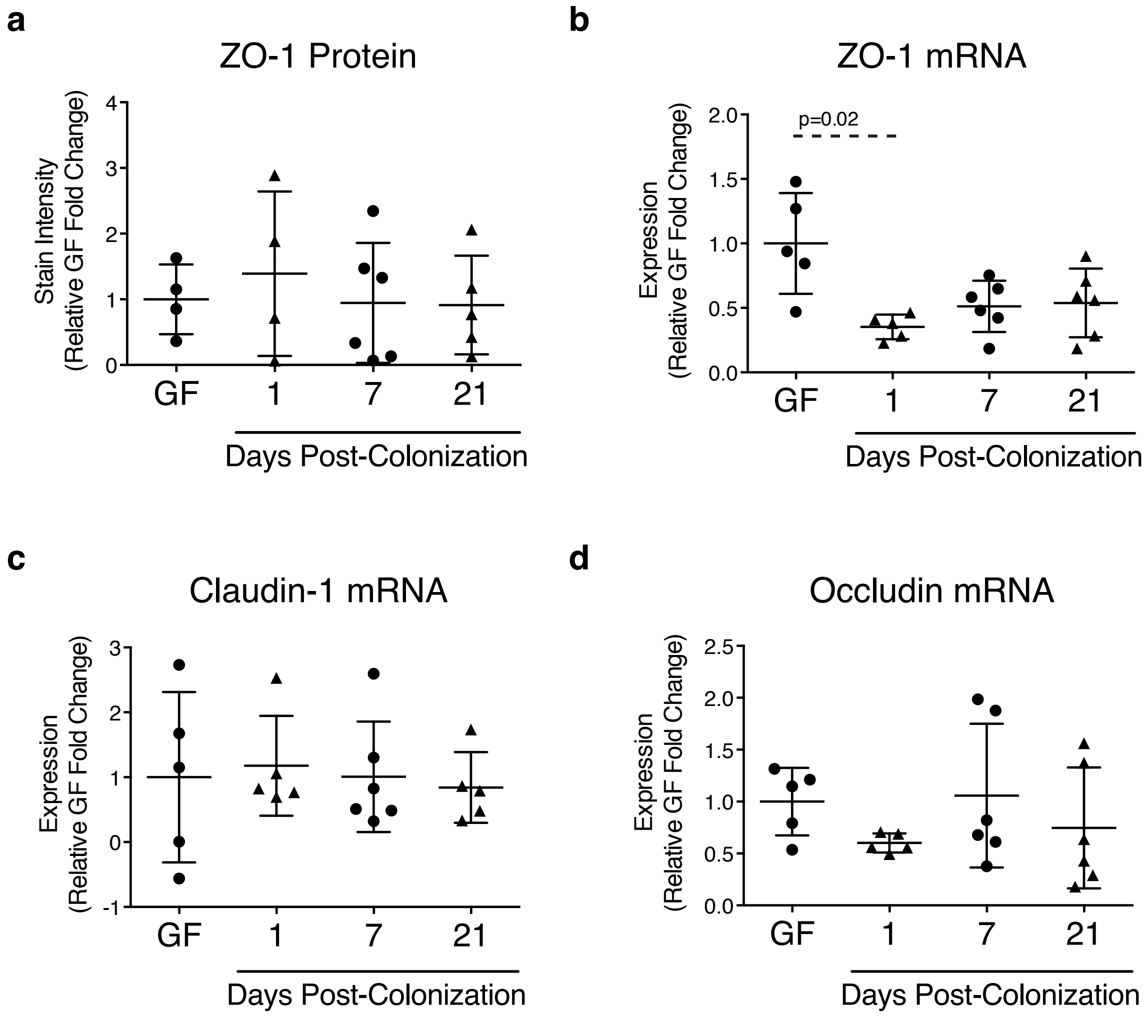

Supplementary Figure S6. Tight junction gene and protein expression post-colonization. Tight junctions were evaluated in germ-free (GF) mice and at days 1, 7, and 21 post-colonization with human fecal microbiota. (a) Immunofluorescent staining was used to assess ZO-1 protein expression. Real-time qPCR was used to evaluate expression of (b) ZO-1 (c) claudin-1 and (d) occludin mRNA. Each data point represents one mouse; the horizontal line and whiskers depict the mean±standard deviation. Significance was assessed using by Kruskal-Wallis test with Dunn's post-hoc test.

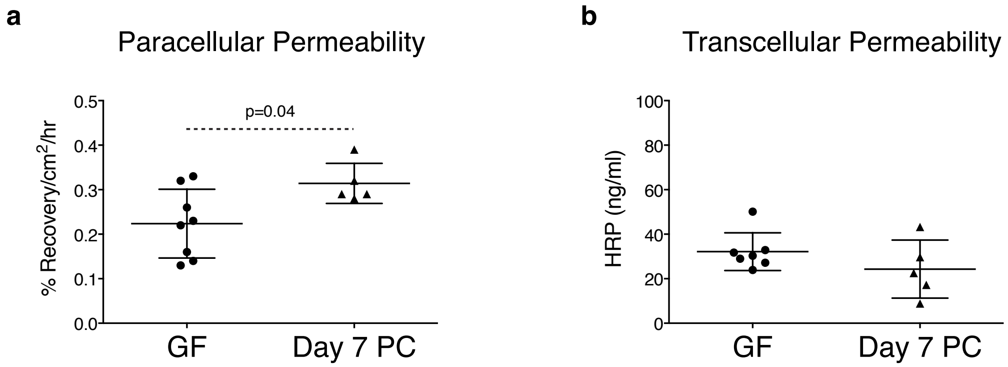

Supplementary Figure S7. Paracellular permeability changes by day 7 post-colonization are independent of colonization mode.

(a) Paracellular and (b) transcellular permeability to ^{51}Cr-EDTA and horseradish peroxidase, respectively, were evaluated by Ussing chambers in germ-free (GF) mice and at day 7 post-colonization (PC) by application of human fecal microbiota to face and paws. Each data point represents one mouse; the horizontal line and whiskers depict the mean±standard deviation. Significance was determined using an unpaired, one-tailed Mann-Whitney test.

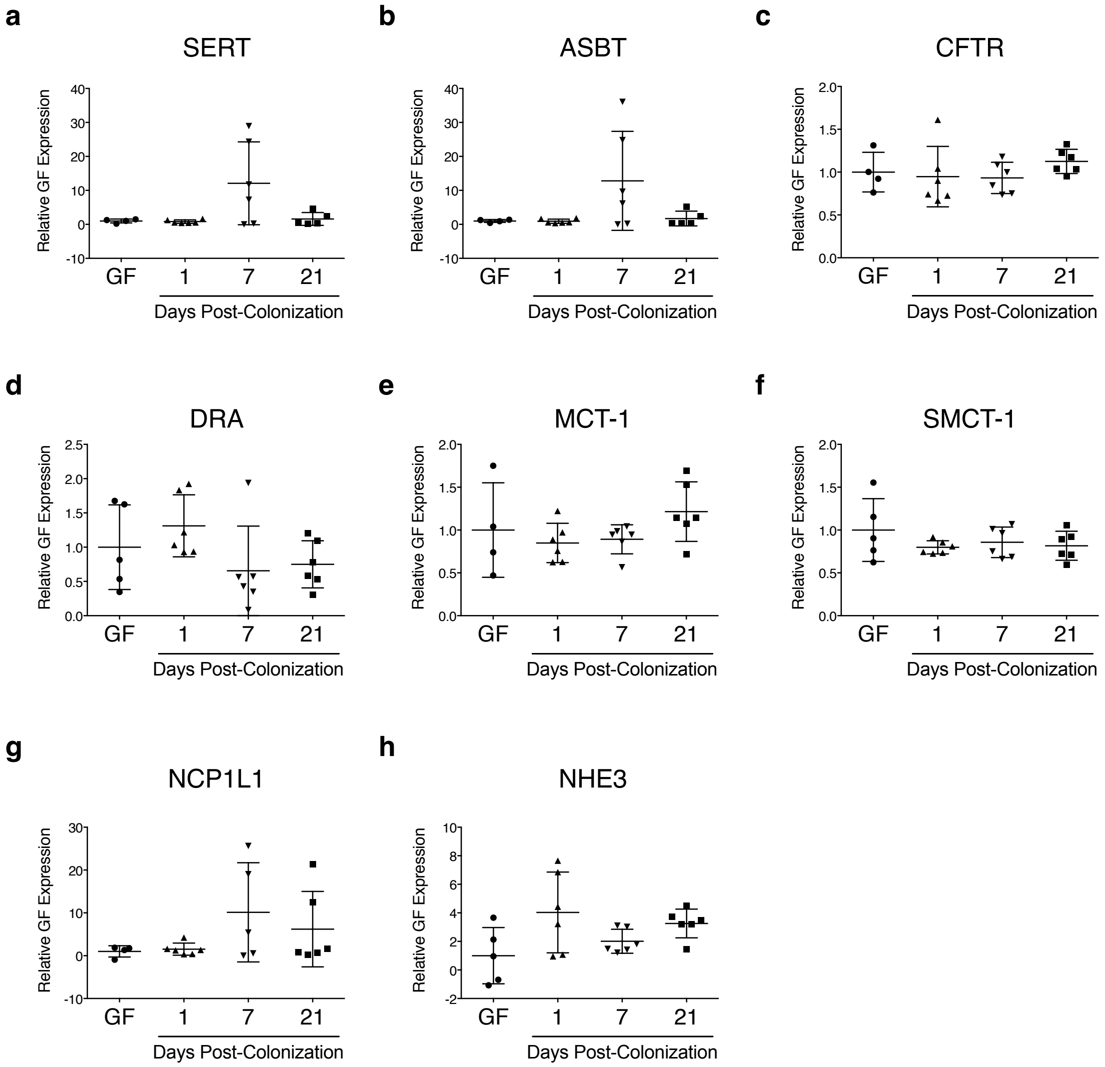

Supplementary Figure S8. Colonic mRNA expression of apical membrane protein transporters are not significantly changed post-colonization.

Real-time qPCR was used to evaluate expression of apical membrane protein transporters in the colon at day 1, 7 and 21 post-colonization with human fecal microbiota of germ-free (GF) mice. (a) SERT (b) ASBT (c) CFTR (d) DRA (e) MCT-1 (f) SMCT-1 (g) NCP1L1 (h) NHE3. Each data point represents one mouse; the horizontal line and whiskers depict the mean±standard deviation. Significance was assessed by Kruskal-Wallis test with Dunn's post-hoc test.

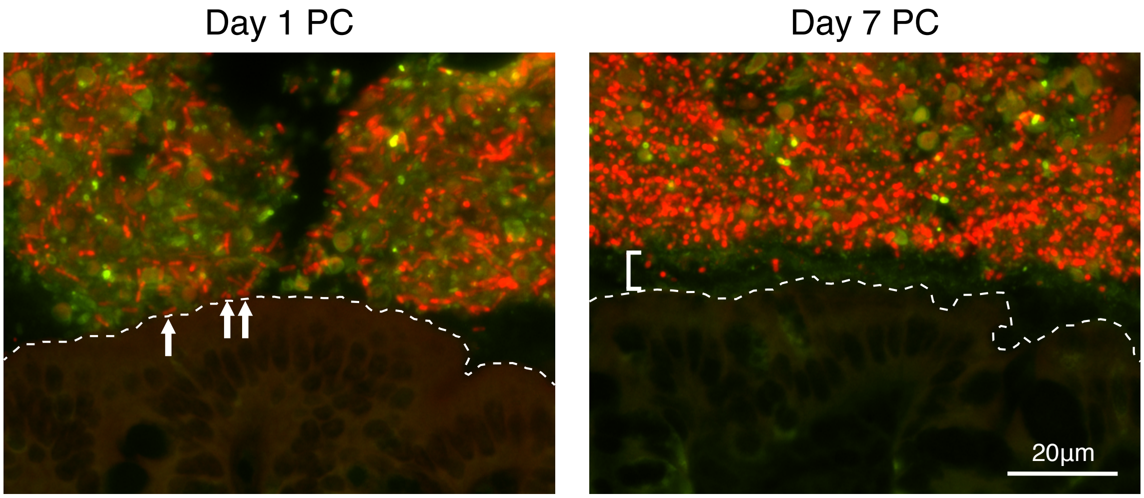

Supplementary Figure S9. Bacterial distribution in the mucus of the proximal colon. Distribution of bacteria was assessed in the proximal colon at days 1 and 7 post-colonization (PC) by fluorescence *in situ* hybridization and immunofluorescent staining using the bacterial 16S rRNA gene probe EUB338 and an anti-mucin-2 antibody, respectively. The dotted lines designate the apical border of the epithelium, white arrows indicate where bacteria are in direct contact with the epithelium, and the white bracket designates the well-defined inner mucus layer.

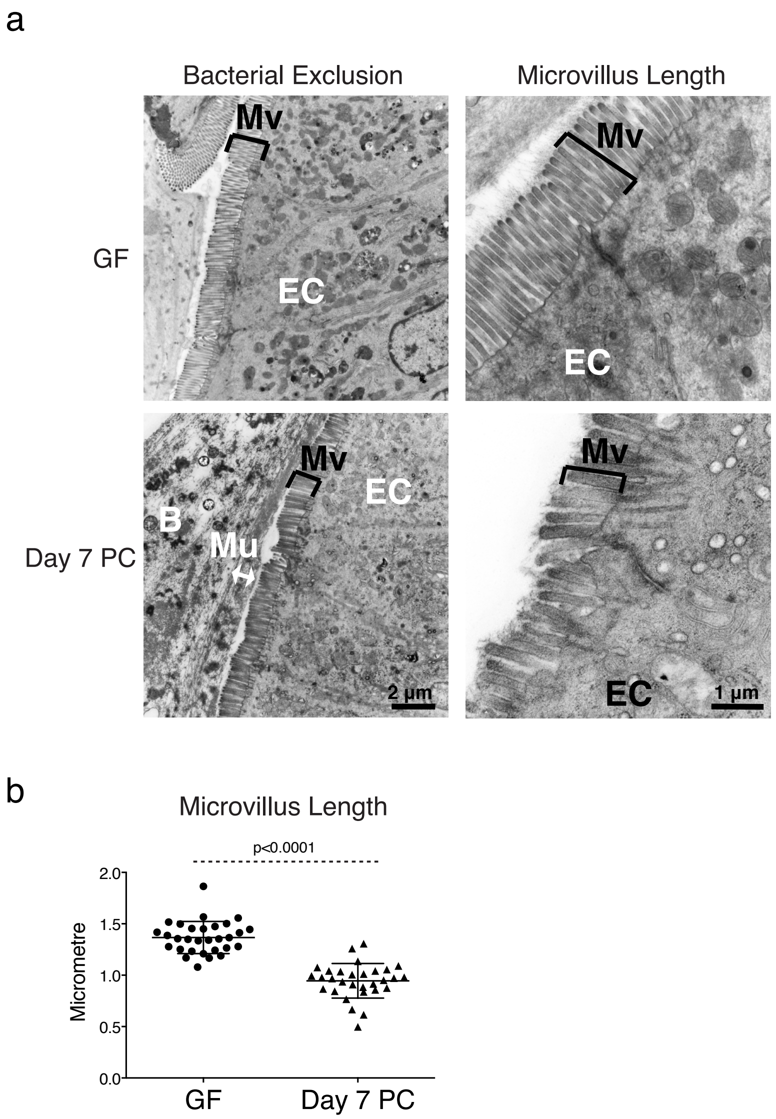

Supplementary Figure S10. Induction of colon structural changes by day 7 post-colonization is independent of colonization mode.

Colon structure was evaluated in germ-free (GF) mice and at day 7 post-colonization (PC) by application of human fecal microbiota to face and paws. (a) Representative electron microscopy images demonstrating bacterial exclusion and microvillus length. Markings indicate microvilli (Mv), epithelial cells (EC), and the black arrow indicates separation of bacteria (B) from the epithelium by the mucus (Mu) layer. (b) Microvillus length. Each data point represents analysis of one image; the horizontal line and whiskers depict the mean±standard deviation. Data was collected over 2-5 independently executed experiments. Significance was determined using an unpaired, two-tailed Mann-Whitney test.

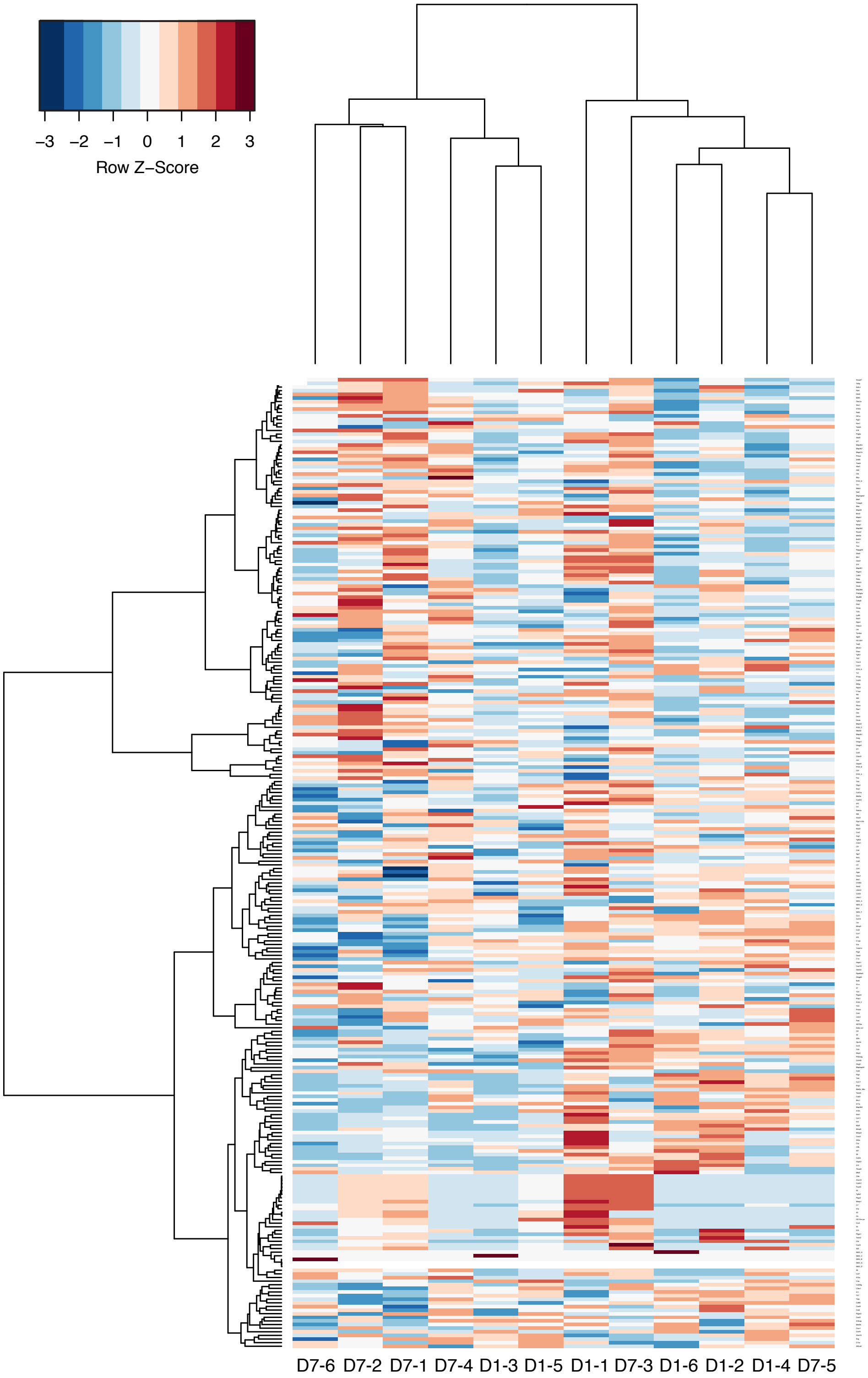

Supplementary Figure S11. Heat map of inflammation-associated gene expression in the colonic IEL-enriched compartment.

IELs from the colon were isolated at days 1 and 7 (D1 and D7, respectively followed by sample number 1-6) post-colonization and Log2 transformed gene expression values of inflammation-associated genes determined by NanoString.

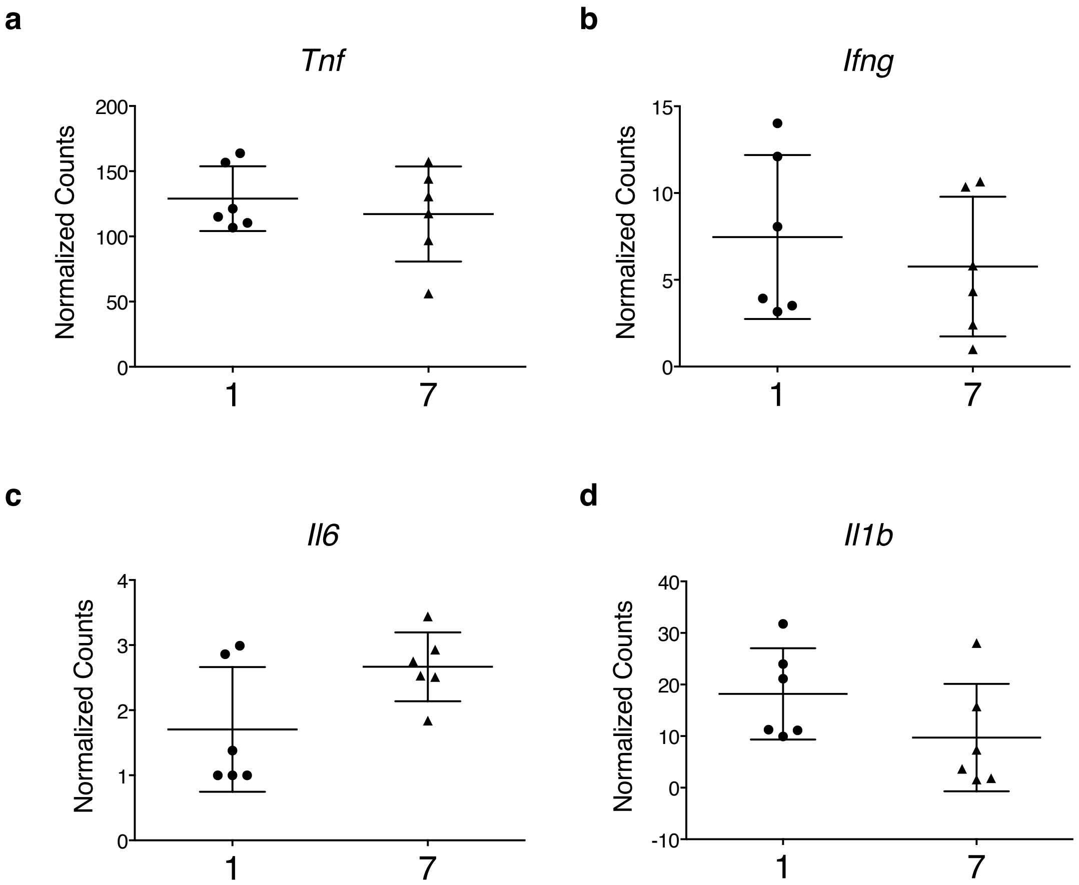

Supplementary Figure S12. Expression of cytokine genes in the colonic IEL-enriched compartment at days 1 and 7 post-colonization.

Cytokine gene expression in the IEL-enriched compartment of the colon was evaluated by NanoString. (a) *Tnf* (b) *Ifng* (c) *Il6* (d) *Il1b*. Each data point represents one mouse; the horizontal line and whiskers depict the mean±standard deviation. Significance was assessed by an unpaired, two-tailed Mann-Whitney test.
